# Central catalytic domain of BRAP (RNF52) recognizes the types of ubiquitin chains and utilizes oligo-ubiquitin for ubiquitylation

**DOI:** 10.1042/BCJ20161104

**Published:** 2017-09-08

**Authors:** Shisako Shoji, Kazuharu Hanada, Noboru Ohsawa, Mikako Shirouzu

**Affiliations:** 1 Division of Structural and Synthetic Biology, RIKEN Center for Life Science Technologies, 1-7-22 Suehiro-cho, Tsurumi-ku, Yokohama 230-0045, Japan; 2 Program for Drug Discovery and Medical Technology Platforms, RIKEN, 1-7-22 Suehiro-cho, Tsurumi-ku, Yokohama 230-0045, Japan

**Keywords:** BRAP, RNF52, ubiquitin-chain assembly, ubiquitin ligases, ubiquitin oligomer, ubiquitin system

## Abstract

Really interesting new gene (RING)-finger protein 52 (RNF52), an E3 ubiquitin ligase, is found in eukaryotes from yeast to humans. Human RNF52 is known as breast cancer type 1 susceptibility protein (BRCA1)-associated protein 2 (BRAP or BRAP2). The central catalytic domain of BRAP comprises four subdomains: nucleotide-binding α/β plait (NBP), really interesting new gene (RING) zinc finger, ubiquitin-specific protease (UBP)-like zinc finger (ZfUBP), and coiled-coil (CC). This domain architecture is conserved in RNF52 orthologs; however, the domain's function in the ubiquitin system has not been delineated. In the present study, we discovered that the RNF52 domain, comprising NBP–RING–ZfUBP–CC, binds to ubiquitin chains (oligo-ubiquitin) but not to the ubiquitin monomers, and can utilize various ubiquitin chains for ubiquitylation and auto-ubiquitylation. The RNF52 domain preferentially bound to M1- and K63-linked di-ubiquitin chains, weakly to K27-linked chains, but not to K6-, K11-, or K48-linked chains. The binding preferences of the RNF52 domain for ubiquitin-linkage types corresponded to ubiquitin usage in the ubiquitylation reaction, except for K11-, K29-, and K33-linked chains. Additionally, the RNF52 domain directly ligated the intact M1-linked, tri-, and tetra-ubiquitin chains and recognized the structural alterations caused by the phosphomimetic mutation of these ubiquitin chains. Full-length BRAP had nearly the same specificity for the ubiquitin-chain types as the RNF52 domain alone. Mass spectrometry analysis of oligomeric ubiquitylation products, mediated by the RNF52 domain, revealed that the ubiquitin-linkage types and auto-ubiquitylation sites depend on the length of ubiquitin chains. Here, we propose a model for the oligomeric ubiquitylation process, controlled by the RNF52 domain, which is not a sequential assembly process involving monomers.

## Introduction

The modifier protein ubiquitin is structurally conserved in eukaryotic cells; post-translational modification with ubiquitin is known as ubiquitylation or ubiquitination [1–3]. Target proteins can be modified at single or multiple sites with a single ubiquitin (ubiquitin monomer) or ubiquitin chains (oligomer or polymer), and various types of ubiquitin linkages form during ubiquitylation. The ‘ubiquitin code’ directs the fate of target proteins in eukaryotic cells [3–5]. Ubiquitin linkages are peptide bonds formed between the C-terminal glycine of the donor ubiquitin and the lysine or N-terminal methionine (M1) residues in the acceptor ubiquitin molecule. Ubiquitin has seven lysine residues (K6, K11, K27, K29, K33, K48, and K63) that are available to form linkages between the ubiquitin molecules; accordingly, eight types of homotypic ubiquitin chains have been identified. In the ubiquitin system, a set of three classes of enzymes, E1 ubiquitin-activating enzymes, E2 ubiquitin-conjugating enzymes, and E3 ubiquitin ligases (hereafter referred to as E1s, E2s, and E3s, respectively), conduct ubiquitylation; E3s catalyze the final step of the ubiquitylation cascade, promoting the transfer of ubiquitin from an E2 to the substrate target and to E3 itself (auto-ubiquitylation). Three distinct major E3 domain families have been identified: RING (really interesting new gene) E3s that include CRL (a complex consisting of a cullin family or a scaffold protein subunit and a RING subunit), HECT (homologous to E6AP carboxyl terminus) E3s, and RBR E3s (containing a RING-in-between-RING domain) [6,7].

RING-finger protein 52 (RNF52), a type of E3, is broadly expressed in eukaryotes from yeast to humans [8]. The human ortholog of RNF52 is BRAP or BRAP2 (BRCA1 [breast cancer type 1 susceptibility protein]-associated protein 2). BRAP reportedly acts as a negative regulator of nuclear import for cyclin-dependent kinase inhibitor 1 (p21) and viral proteins [9–11]. Moreover, BRAP has been identified as a genetic risk factor for the development of metabolic syndrome and cardiovascular disease, including heart attack [12–14], and is involved in the NF-κB (nuclear factor kappa-light-chain-enhancer of activated B cells) inflammatory cascade [15,16]. BRAP is also known as IMP (impedes mitogenic signal propagation) because it acts as a modulator in Ras-MAPK (mitogen-activated protein kinase) signaling [8,16,17] and the Raf (rapidly accelerated fibrosarcoma kinase)/MEK (MAPK kinase)/ERK (extracellular signal-regulated kinase) cascade that is related to IFN (interferon)-γ production in T-cells [18]. Although BRAP was initially identified as a protein that binds to the nuclear localization signal of the tumor suppressor BRCA1 [19], BRAP actually interacts with a variety of proteins [20].

The central catalytic core region of BRAP has four subdomains, namely an NBP (nucleotide-binding α/β plait) motif, a RING zinc finger motif, a ZfUBP [ubiquitin-specific protease (UBP)-like zinc finger] motif, and a CC (coiled-coil) motif. The domain architecture of this region, encompassing these four subdomains, is especially conserved in RNF52 orthologs (Supplementary Data S1) and so can be referred to as the RNF52 domain. Nevertheless, the precise function of the RNF52 domain, comprising NBP–RING–ZfUBP–CC, remains unknown; thus, the underlying E3 activity of BRAP in the ubiquitin system has not been characterized.

Here, we explored how the RNF52-domain functions with regard to the E3 activity of BRAP in the ubiquitylation process. We report on the characteristics of the RNF52 domain of BRAP with respect to ubiquitin chains (oligoubiquitin) and discuss a novel concept of ubiquitylation, utilizing oligoubiquitin, and catalyzed by the RNF52 domain.

## Experimental

### Accession numbers corresponding to online databases

**Figure 1.**
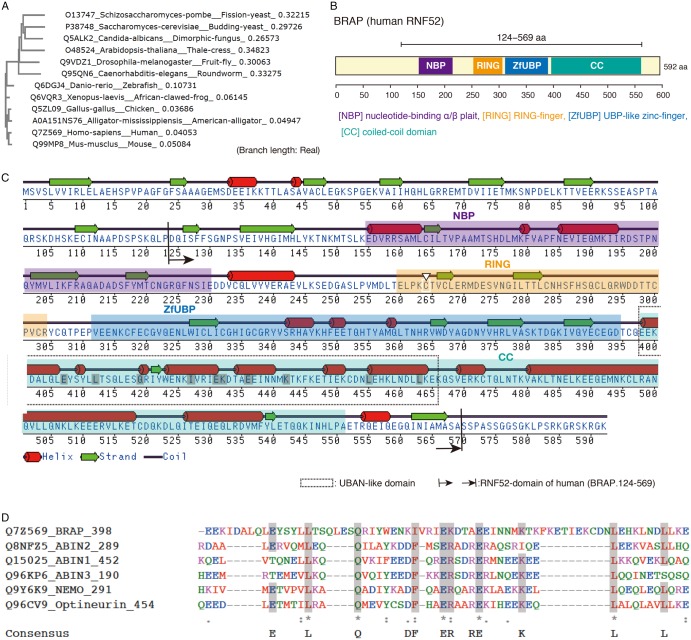
Characteristics of amino acid sequences of BRAP/RNF52. (**A**) Phylogenetic tree of RNF52 amino acid sequences in eukaryotes, including mammals (*Homo sapiens* and *Mus musculus*), a bird (*Gallus gallus*), a reptile (*Alligator mississippiensis*), an amphibian (*Xenopus laevis*), a fish (*Danio rerio*), a plant (*Arabidopsis thaliana*), an insect (*Drosophila melanogaster*), a worm (*Caenorhabditis elegans*), and fungi (*Candida albicans*, *Saccharomyces cerevisiae*, and *Schizosaccharomyces pombe*); the tree was generated using the Clustal Omega program 1.2.1 (http://www.ebi.ac.uk/Tools/msa/clustalo/). UniProtKB accession codes are given before the species name. The amino acid sequences and multiple sequence alignment are shown in Supplementary Data S1. (**B**) Schematic diagram showing the conserved central catalytic domain (RNF52 domain) of BRAP; this domain contains four subdomains that are highly conserved in vertebrates: NBP, nucleotide-binding α/β plait; RING, really interesting new gene zinc finger; ZfUBP, ubiquitin-specific protease (UBP)-like zinc finger; CC, coiled-coil domain. (**C**) Predicted secondary structure and amino acid sequence of BRAP including the region at amino acid positions 124–569 that corresponds to the RNF52 domain. Inter Pro (http://www.ebi.ac.uk/interpro/) was used for the motif and domain searches. The program DSC (Discrimination of Protein Secondary Structure Class) [49] was used to predict secondary structures. The white downward-pointing triangle indicates the RING-finger motif mutation (C264). (**D**) Sequence alignment of a part of the BRAP-CC domain and the UBAN domains of human ABIN proteins (ABIN-1, ABIN-2, ABIN-3, NEMO, and optineurin). The highly conserved amino acids in the UBAN motif [50] are shaded in gray.

The approved HGNC [human genome organization (HUGO) Nomenclature Committee] name and UniProtKB (UniProt Knowledgebase) ID numbers are listed as follows: UBC (ubiquitin), P0CG48; UBA1 [also known as UBE1 (ubiquitin-activating enzyme E1)], P22314; UBE2D1 (also known as UbcH5A), P51668; and BRAP (also known as human RNF52), Q7Z569. The accession numbers for the RNF52 orthologs from other species are shown in Figure 1A and Supplementary Data S1.

### Construction of recombinant plasmid vector for gene expression

For recombinant protein expression using *Escherichia coli*, and for cell-free synthesis-coupled transcription–translation, the target cDNA fragments were sub-cloned into the pCR2.1-TOPO vector (Thermo Fisher Scientific, Waltham, MA, U.S.A.). Proteins, subjected to affinity purification, were N-terminally fused with a modified natural poly-histidine N11-tag (amino acid sequence: MKDHLIHNHHKHEHAHAEH) or a maltose-binding protein (MBP) tag, as well as with a TEV (tobacco etch virus) protease recognition site and a GSSGSSG linker sequence [21]. These tags were introduced using TOPO cloning. In the cell-free synthesis system, proteins, tagged with either the N11-tag or the MBP tag, were designed to have the same protein sequence if the tag was to be removed using TEV. For baculovirus-insect cell expression, we used the Bac-to-Bac Baculovirus Expression System (Thermo Fisher Scientific).

cDNAs were derived from the original clone sets as follows: ubiquitin from a clone set obtained from the Institute of Medical Science, the University of Tokyo; UBE2D1 from a clone set purchased from Thermo Fisher Scientific; UBA1 from a clone set purchased from OriGene Technologies (Rockville, MD, U.S.A.); and BRAP from a clone set obtained from the Kazusa DNA Research Institute (Kisarazu, Chiba, Japan).

### Protein expression and purification

Ubiquitin, di-, tri-, and tetra-ubiquitin chains (Ub2, Ub3, and Ub4) were expressed in the *E. coli* strain KRX (Promega Corporation, Madison, WI, U.S.A.), which had been pre-cultured in Luria-Bertani medium at 37°C. Protein expression was induced by adding 0.2% (w/v) l-rhamnose, and cells were incubated at 25°C for 20 h. Ubiquitin and ubiquitin chains (oligo-ubiquitin) were purified according to the method of Pickart and Raasi [22]. M1-linked ubiquitin chains, and their phosphomimetic (S65D) mutants, were synthesized in our laboratory (RIKEN CLST Division of Structural and Synthetic Biology). The other ubiquitin monomer mutants and K6-, K11-, K27-, K29-, K33-, K48-, and K63-linked di-ubiquitins (Ub2) were purchased from R&D systems (Minneapolis, MN, U.S.A.).

T7 promoter-driven expression and cell-free synthesis of N-terminal affinity-tagged N11-BRAP (124–569), MBP-BRAP (1–592, full-length), and N11-UBE2D1 were performed in an *E. coli* S30 extract in S30 buffer [10 mM Tris acetate buffer at pH 8.2, containing 60 mM potassium acetate, 16 mM magnesium acetate, and 1 mM dithiothreitol (DTT)] [23].

The N-terminal affinity-tagged recombinant BRAP (124–569), BRAP (1–592), and a RING-finger motif mutant (C264S), in which the first cysteine residue in the RING-finger consensus sequence was replaced with a serine residue [8,16,17], were synthesized using a zinc ion supplemented cell-free protein expression system [24] and then purified using an AKTA 10S system (GE Healthcare, Aurora, OH, U.S.A.) with a HisTrap or MBPTrap HP column (GE Healthcare); the AKTA 10S system was washed with a concentration gradient buffer (50 mM Tris–HCl buffer at pH 8.0, containing 1 M NaCl and 10 mM imidazole). The N11-tagged recombinant proteins were eluted with a concentration gradient of imidazole (from 10 to 500 mM) in elution buffer (50 mM Tris–HCl buffer at pH 8.0, containing 0.5 M NaCl). MBP-tagged full-length BRAP was eluted with a concentration gradient of maltose (from 0 to 5 mM) in elution buffer (20 mM Tris–HCl buffer at pH 7.5, containing 0.2 M NaCl and 1 mM DTT). Imidazole or maltose was removed by overnight dialysis at 4°C in wash buffer. The affinity-tags were removed by incubation at 4°C for 20 h with TEV protease. The resulting tag-cleaved proteins were purified by ion exchange chromatography and gel filtration with the final buffer [20 mM 4-(2-hydroxyethyl)-1-piperazineethanesulfonic acid (HEPES)–NaOH buffer at pH 7.5, containing 150 mM NaCl] using HiLoad 16/600 Superdex columns (GE Healthcare). His6-tagged UBA1 was expressed in insect Sf9 cells using the Bac-to-Bac Baculovirus Expression System (Thermo Fisher Scientific) and purified according to the method of Huang et al. [25].

Purified protein concentrations were determined by measuring the absorbance at 280 nm using a NanoDrop spectrophotometer (Thermo Fisher Scientific).

### Ubiquitylation assay

The *in vitro* ubiquitylation assay of the RNF52 domain of BRAP was performed in a 25-µl reaction mixture containing 20 mM HEPES–NaOH at pH 7.5, 2 mM MgCl_2_, 50 nM UBA1 (for E1 activity), 0.5 µM UBE2D1 (for E2 activity), 1 µM BRAP (for E3 activity), 10 µM ubiquitin or ubiquitin chains, and 5 mM ATP (adenosine triphosphate). The reaction mixtures without ATP were preincubated on ice for 20 min; thereafter, the ubiquitylation reaction was initiated by adding ATP followed by incubation at 37°C for 60 or 90 min. To screen for BRAP activity in 34 different human E2s, we used E2^scan^ Kit version 2 (Ubiquigent, Dundee, U.K.). The reactions were stopped by adding 20 µl of 2.5× SDS–PAGE (sodium dodecyl sulfate–polyacrylamide electrophoresis) sample loading buffer [5% (w/v) SDS, 250 mM DTT, 15% (v/v) glycerol, 140 mM Tris–HCl at pH 6.8, and 0.01% (w/v) bromophenol blue] and incubated at 95°C for 3 min.

The ubiquitylation reactions were resolved by SDS–PAGE on a 10–20% gradient gel and visualized by Coomassie Brilliant Blue (CBB) G-250 staining. For quantification of the protein band signals, the CBB-stained gel images were acquired using the LAS-3000 imaging system (GE Healthcare) with white light transillumination. The reaction products were also detected by western blot (WB) analysis using an anti-BRAP clone D-5 (Santa Cruz Biotechnology, Dallas, TX, U.S.A.), which is a monoclonal antibody raised against amino acids 41–340 of BRAP and anti-multi Ub antibody clone FK2 (Medical and Biological Laboratories, Nagoya, Aichi, Japan), which recognizes both mono- and poly-ubiquitinylated species but not free ubiquitin. The WB signals for auto-ubiquitylated or non-ubiquitylated BRAP (124–569) were detected using an Immobilon Chemiluminescence Detection Kit (Merck KGaA, Darmstadt, Germany), and the luminescent signals were acquired using the luminescent image analyzer in LAS-3000. The protein band signals from CBB-stained gels, and from WBs using the anti-BRAP antibody (clone D-5), were quantified using the ImageQuant TL software (GE Healthcare).

For mass spectrometry analyses, gel regions, containing proteins, were excised and digested with trypsin (Merck KGaA) for 20 h at 37°C. The resulting peptides were analyzed by liquid chromatography–electrospray ionization tandem mass spectrometry (LC–ESI-MS/MS) at the RIKEN BSI Research Resources Center.

### Protein–protein interaction assay

SPR (surface plasmon resonance) sensorgrams, showing the profiles of protein–protein interactions, were obtained from ligand-binding experiments based on purified recombinant proteins (Supplementary Data S2), using the Biacore 3000 system (GE Healthcare). For the Biacore assays, we immobilized the ligand to a CM5 sensor chip (GE Healthcare) using an Amine Coupling Kit (GE Healthcare) under the following conditions: for activation, 0.4 M 1-ethyl-3-(3-dimethylaminopropyl)-carbodiimide and 0.1 M *N*-hydroxysuccinimide; for immobilization, sodium acetate–acetic acid buffer solutions at pH 5.0 for ubiquitin chains and at pH 4.5 for the ubiquitin monomer; for deactivation, 1 M ethanolamine–HCl at pH 8.5. Mobile molecules (analytes) were serially diluted in running buffer [10 mM HEPES–NaOH at pH 7.5, 150 mM NaCl, 0.005% (v/v) Biacore surfactant P20]. The buffer was filtered with a 0.22-µm vacuum filter and degassed prior to use. All experiments were performed at 25°C. The SPR sensorgrams, obtained for the control flow cell, were subtracted from the data for the flow cell immobilized with a ligand. Protein–protein interaction experiments, using monomer ubiquitin and M1-linked and K63-linked di-ubiquitin chains with BRAP (124–569) (RNF52 domain) or BRAP (1–592) (full-length), were repeated at least three times. The experiments using other ubiquitin linkages were performed once using full-length and the RNF52 domain, respectively. The binding affinity and interaction kinetics in each experiment were obtained from Biacore sensorgrams using the BIAevaluation software version 4.1.1 (GE Healthcare) according to its instruction manual.

## Results

### Binding preference of the RNF52 domain of BRAP for ubiquitin-linkage types

Because the domain architecture of BRAP contains ZfUBP–CC motifs (Figure 1B,C), and considering the findings discussed below, we hypothesized that BRAP recognizes the chain structure of linear ubiquitin. The ZfUBP (also called ZnF UBP) motif is found in a subfamily of UBPs [26,27]; reportedly, a member of this subfamily, CYLD, specifically cleaves M1- and K63-linked ubiquitin chains [28]. Compared with K48-linked di-ubiquitin, which forms a tight dimer, both M1-linked and K63-linked di-ubiquitin chains adopt a more relaxed conformation; however, the spatial structures of the K63-linked chain and the M1-linked chain are directionally different from each other [29,30]. A well-known M1-linked ubiquitin-chain-binding protein, NF-κB essential modulator (NEMO) which is an essential modulator of NF-κB, contains a CC motif as its ubiquitin-binding domain [31] and a zinc finger, arranged sequentially [32]. We found that the consensus amino acid sequence motif of the UBAN domain, found in human A20-associating cytosolic (ABIN) proteins (ABIN-1, ABIN-2, ABIN-3, NEMO, and optineurin), was also found within the BRAP-CC motif (Figure 1D).

Based on these observations, we first examined whether the RNF52 domain of BRAP recognizes ubiquitin monomers and/or ubiquitin chains. In the first experiment, we used K6-, K11-, K27-, K29-, K33-, K48-, K63-, and M1-linked di-ubiquitin chains (hereafter termed K6-Ub2, K11-Ub2, K27-Ub2, K29-Ub2, K33-Ub2, K48-Ub2, K63-Ub2, and M1-Ub2, respectively) to examine whether the RNF52 domain of BRAP binds to all linkage types of oligo-ubiquitin. To perform this protein–protein interaction assay, we used purified protein samples of monomeric ubiquitin, ubiquitin chains, and a fragment containing the entire RNF52 domain of BRAP [amino acids 124–569; referred to as BRAP (124–569)] (Supplementary Data S2). The RNF52 domain location of BRAP was determined based on multiple amino acid sequence alignment of RNF52 orthologs (Supplementary Data S1) and predictions for protein secondary structure (Figure 1C).

**Figure 2.**
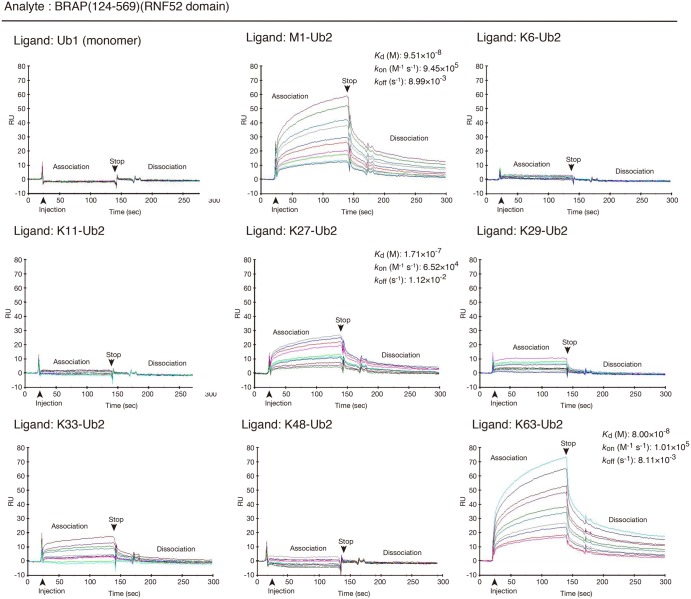
Protein–protein interaction profiles showing the binding of the RNF52 domain of BRAP to ubiquitin monomers and to all di-ubiquitin oligomer (Ub2) linkage types. Surface plasmon resonance sensorgrams from the Biacore assay (i.e. protein–protein interaction profiles) are shown. Resonance signal, expressed as the response unit (RU), indicates the degree of binding between BRAP and the various ubiquitin molecules. The immobilized ligands (i.e. ubiquitin and the various di-ubiquitin oligomers) are shown in the top left of each panel. The analyte (applied as the mobile molecule to an immobilized ligand on the sensor chip) was BRAP (124–569) (RNF52 domain). The measured equilibrium dissociation constant (*K*_d_), association rate constant (*k*_on_), and dissociation rate constant (*k*_off_), for each binding, are shown in the top right of each panel where possible.

Biacore sensorgrams (protein–protein interaction profiles) (Figure 2) showed that BRAP (124–569) did not bind to the ubiquitin monomers (Ub1), K6-Ub2, K11-Ub2, or K48-Ub2, whereas it did bind to K27-Ub2, K63-Ub2, and M1-Ub2. In this experiment, the *K*_d_ (9.51 × 10^−8^ M) and the *k*_off_ (8.99 × 10^−3^ s^−1^) of BRAP for M1-Ub were not significantly different from the *K*_d_ (8.00 × 10^−8^ M) and *k*_off_ (8.11 × 10^−3^ s^−1^) for K63-Ub. Compared with the *k*_on_ (9.45 × 10^−5^ M^−1^ s^−1^) for M1-Ub2, the *k*_on_ (1.01 × 10^−5^ M^−1^ s^−1^) for K63-Ub was slightly higher for K63-Ub2 than for M1-Ub2. The binding affinity of BRAP (124–569) for K27-Ub2 was weaker than that for M1-Ub2 and K63-Ub2. In this experiment, the Biacore sensorgrams indicated that BRAP (124–569) may be bound to K29-Ub2 and K-33-Ub2; however, the response signals were too weak to determine binding affinity.

**Figure 3.**
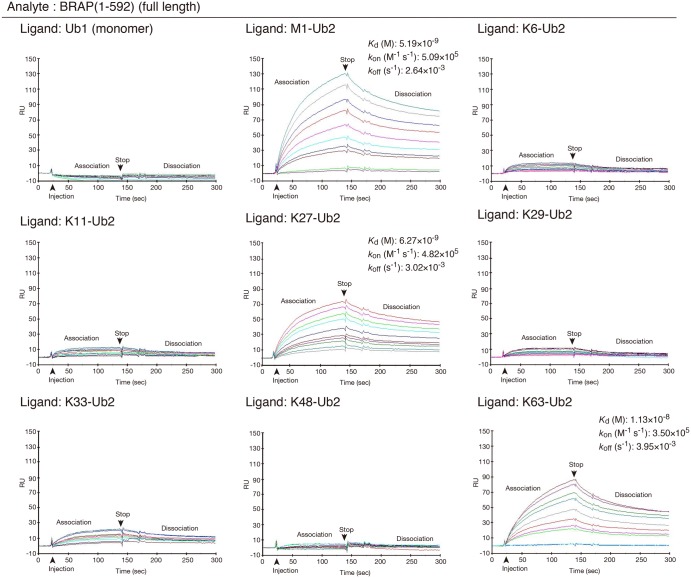
Protein–protein interaction profiles showing the binding ability of full-length BRAP against ubiquitin monomers and all ubiquitin-linkage types of di-ubiquitin chains (Ub2). Surface plasmon resonance sensorgrams from the Biacore assay (i.e. protein–protein interaction profiles) are shown. Resonance signal, expressed as the response unit (RU), indicates the degree of binding between BRAP and the various ubiquitin molecules. The immobilized ligands (i.e. ubiquitin and the various di-ubiquitin oligomers) are shown in the top left of each panel. The analyte (applied as the mobile molecule to an immobilized ligand on the sensor chip) was BRAP (1–592) (full-length). The measured equilibrium dissociation constant (*K*_d_), association rate constant (*k*_on_), and dissociation rate constant (*k*_off_), for each binding, are shown in the top right of each panel where possible.

We next examined the binding preference of full-length BRAP (1–592) for different ubiquitin-linkage types (Figure 3). The binding of full-length BRAP for all the tested ubiquitin-linkage types was nearly the same as that for BRAP (124–569), although the binding affinity was stronger than that for BRAP (124–569). We conclude that the RNF52 domain is responsible for recognizing the linkage type of the ubiquitin chain.

### Identification of the E2 partner of BRAP

BRAP (124–569) bound to the ubiquitin chain (oligo-ubiquitin) but not to the ubiquitin monomer. We then examined whether BRAP (124–569) can ligate oligo-ubiquitin in a ubiquitylation reaction. To find E2s that enable the E3 activity of BRAP (Supplementary Data S3A), we first screened 34 human E2s using purified protein components in a ubiquitylation reaction. Combining BRAP with UBE2D1, UBE2D2, UBE2D3, or UBE2D4 efficiently produced polyubiquitin chains, whereas combining BRAP with the other E2s failed to generate polyubiquitin chains. We also performed the same *in vitro* ubiquitylation assay in the presence of a RING-finger motif mutant (RING-finger defective mutant) of BRAP (C264S) and confirmed that these polyubiquitylation reactions were dependent on the activity of BRAP. These results clearly show that the most suitable E2 partners of BRAP, for ubiquitin-to-ubiquitin ligation, were members of the UBE2D family (also known as the UbcH5 family for human homologs of yeast UBC4/5). UBE2D proteins are Class I E2s and have a bare UBC fold [33]; however, other Class I E2s we examined (i.e. UBE2A and UBE2B) could not promote polyubiquitylation when combined with BRAP. Therefore, BRAP recognizes UBE2D family proteins rather than other Class I E2s.

UBE2H (homolog to yeast UBC8, E2-20K) is reported to bind BRAP using the yeast two-hybrid system [34]; however, the combination of UBE2H with BRAP did not catalyze ubiquitylation *in vitro*. Additionally, we examined whether BRAP binds to UBE2D1. Biacore sensorgrams indicated that both BRAP (1–592) (full-length) and BRAP (124–569) (RNF52 domain) did not bind to UBE2D1 (Supplementary Data S3B). Accordingly, we conclude that for ubiquitin ligation to occur, it is important that the binding affinity of E2 for E3 (UBE2D1 for BRAP, in the present study) is weak rather than strong.

### Ubiquitin-linkage types generated by combining UBE2D1 and the RNF52 domain of BRAP with monomeric ubiquitin

Based on previous data, we used UBE2D1 to represent the UBD2D family as an E2 partner for the RNF52 domain of BRAP, BRAP (124–569), in subsequent experiments.

**Figure 4.**
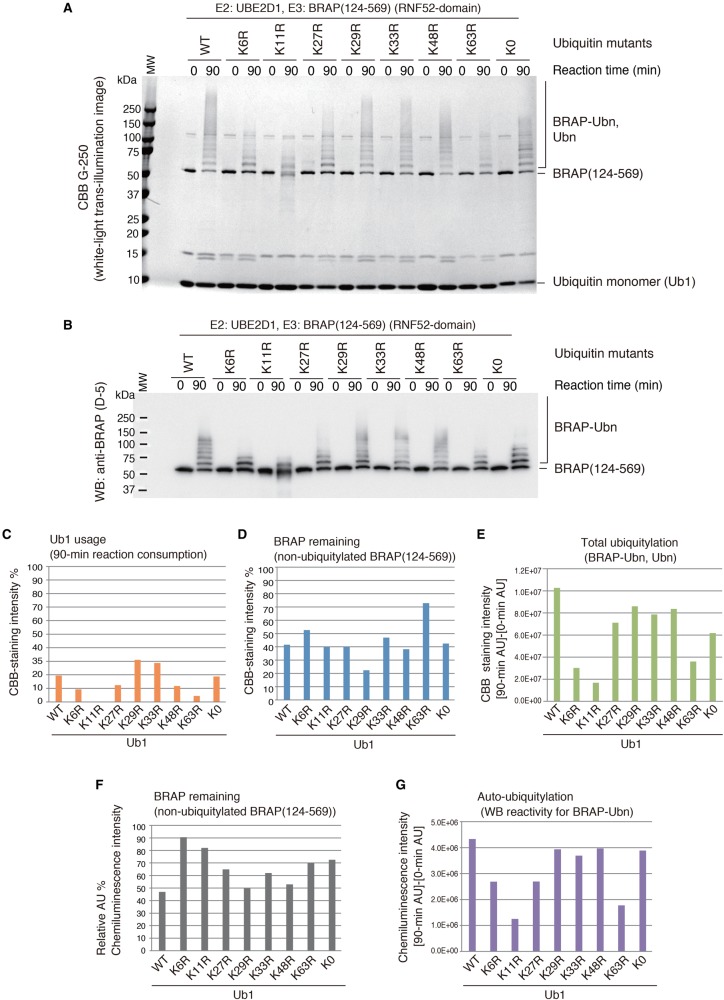
The E3 activity of the RNF52 domain of BRAP on ubiquitin monomer and its different lysine mutants. Ubiquitylation assays of the BRAP (124–569) fragment (RNF52 domain of BRAP) in the presence of UBA1, UBE2D1, and WT ubiquitin or the ubiquitin mutants: K6R, K11R, K27R, K29R, K33R, K48R, K63R, and K0 (no lysine residue). (**A**) SDS–PAGE gels of the ubiquitylation reaction products stained with CBB G-250. (**B**) WB of the same samples as in (**A**) probed with an anti-BRAP antibody (clone D-5). (**C**–**G**) The band signals from the CBB-stained gels and WB were quantified using the ImageQuant TL software. (**C**) Ub1 (ubiquitin monomer) usage was calculated by determining what percentage of the band had disappeared after a 90-min reaction, assuming that the band signal of the unshifted bands for the Ub1 in each lane at time 0 (zero) of the reaction was 100%. (**D** and **F**) The percentage of BRAP (124–569) remaining at the end of the reaction (i.e. the non-ubiquitylated BRAP present after a 90-min reaction) was calculated by assessing band signal intensity that corresponded to non-ubiquitylated BRAP (124–569) in each lane after a 90-min reaction, assuming that the band signal for the unshifted Ub1 bands in each lane, at reaction time 0, was 100%. (**E**) To assess total ubiquitylation, the intensity of the high molecular mass signals, detected in the CBB-stained gel after a 90-min reaction, was obtained, and the signals from the same area of the gel at reaction time 0 were subtracted as background. (**G**) To assess the production of auto-ubiquitylated BRAP, the signals detected in the high molecular mass area of the WB, after a 90-min reaction, were obtained, and the signals from the same area of the blot at reaction time 0 were subtracted as background. AU, arbitrary unit of the band signal intensity. These graphs show the average values obtained from two independent experiments.

Before performing the ubiquitylation assay using oligo-ubiquitin (ubiquitin chains), we examined the types of ubiquitin linkage, generated by the E2–E3 combination of UBE2D1 and BRAP (124–569), using an *in vitro* ubiquitylation assay in the presence of different ubiquitin monomers; the monomers used were wild-type (WT) and a series of ubiquitin lysine (K) mutants: K6R, K11R, K27R, K29R, K33R K48R, K63R, and K0 (a ubiquitin mutant with all K residues replaced by R; i.e. no K) (Figure 4). Ubiquitylation efficiency was decreased in the *in vitro* assay using all of these ubiquitin K-R mutants, indicating that all types of internal K-linked ubiquitin chains were generated by the combination of UBE2D1 and BRAP (124–569). Failed ubiquitylation in the cases of K6R, K11R, and K63R were especially notable (Figure 4E).

We also conducted the same *in vitro* ubiquitylation assay in the presence of the S65D ubiquitin mutant, methylated ubiquitin (Me), K6-only, and K63-only ubiquitin mutants (Supplementary Data S4). The S65D ubiquitin mutant, in which a serine residue (a target of kinases) was replaced with an aspartic acid residue in a phosphomimetic mutation, was based on a recent study, indicating that ubiquitin, phosphorylated at S65, affects ubiquitin-chain elongation and increases ubiquitin-chain hydrolysis [35]. Ubiquitylation was slightly reduced in the reaction using the S65D ubiquitin mutant. Methylated ubiquitin is chemically modified to block all of its free amino groups; thereby, it does not form polyubiquitin chains, but can ligate protein substrates. Ubiquitylation was found to occur in the reaction using methylated ubiquitin. The results of WB analysis, using an anti-BRAP antibody, indicated that auto-ubiquitylation (i.e. peptide linkage between the glycine residue at the ubiquitin C-terminus and lysine residues on BRAP) had also occurred. In the case of reactions using K6-only and K63-only ubiquitin mutants, the results of WB analysis, using an anti-multi-ubiquitin antibody (FK2), were unclear; however, WB, using an anti-BRAP antibody, indicated that auto-ubiquitylation had occurred at high levels. In two independent experiments, WB, using both anti-BRAP and anti-multi-ubiquitin antibodies, showed that ubiquitylation did not occur for the K6R, K11R, and K63R mutants (upper and lower panels, Supplementary Data S4). These results suggest that the RNF52 domain of BRAP has a preference for ubiquitin linkages involving K6, K11, or K63. Our result for the K63R mutant is also consistent with a previous study on K63-linked chains formed by BRAP-mediated ubiquitylation [36].

Additionally, we explored ubiquitin-linkage types using MS analysis of the monomeric ubiquitylation products (Supplementary Data S5). Peptide mass spectrometry, conducted on a gel slice obtained from a high molecular mass area of SDS–PAGE, detected M1-, K11, and K48-linkages, but not K6 or K63 linkages, or ubiquitylated BRAP fragments, although the results of WB with an anti-BRAP antibody indicated that auto-ubiquitylation had occurred. The results suggest that the E2–E3 combination of UBE2D1 and BRAP (124–569) with a ubiquitin monomer (Ub1) generates M1-, K11-, and K48-linked chains mainly, whereas K6- and K63-linked chains and auto-ubiquitylation may be minor events during monomeric ubiquitylation. It is unlikely that BRAP preferentially catalyzes K48 linkage based on the result from the experiment using the ubiquitin K48R mutant. Because UBE2D1 catalyzes K48 linkage [37], BRAP-recruited Ub1-charged UBE2D1 may be available to drive K48-linked ubiquitylation proactively.

### The RNF52 domain of BRAP directly utilizes intact ubiquitin chains for ubiquitylation

**Figure 5.**
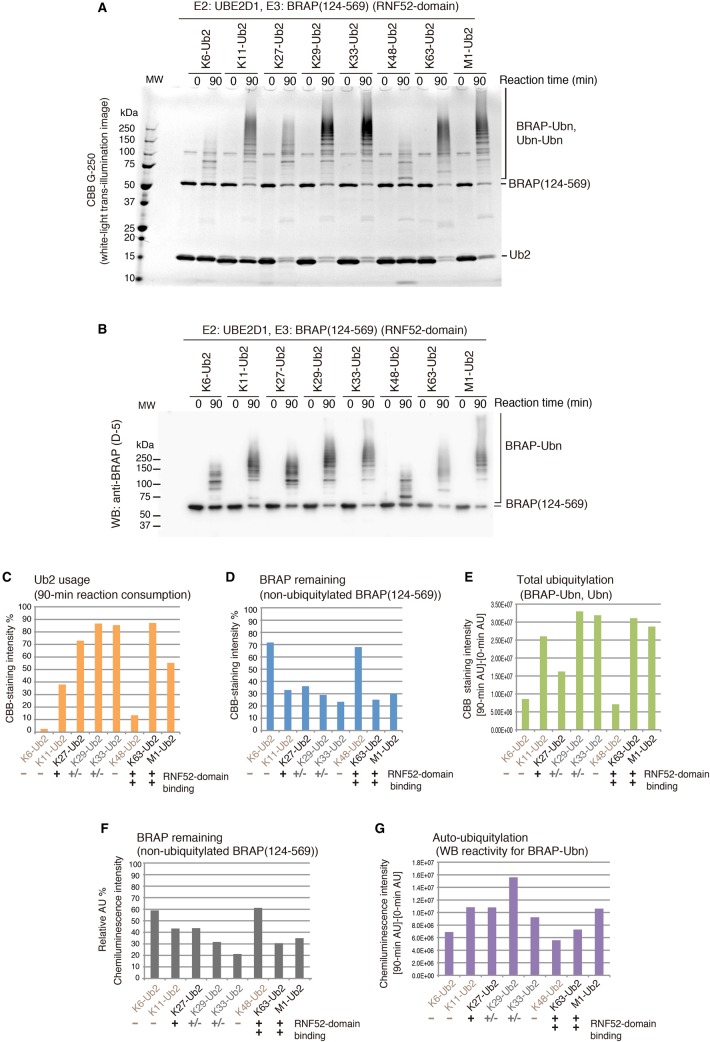
Oligomeric ubiquitylation preference of the RNF52 domain of BRAP for different linkage types of di-ubiquitin chains. Ubiquitylation assays of the BRAP (124–569) fragment (RNF52 domain of BRAP) in the presence of UBA1, UBE2D1, and K6-, K11-, K27-, K29-, K33-, K48-, and K63-linked di-ubiquitin chains (Ub2). (**A**) SDS–PAGE gels of the ubiquitylation reaction products stained with CBB G-250. (**B**) WB of the same samples as in (**A**) probed with an anti-BRAP antibody (clone D-5). (**C**–**G**) The band signals from the CBB-stained gels and WB were quantified using the ImageQuant TL software. (**C**) Ub2 (di-ubiquitin chain) usage was calculated by determining what percentage of the band had disappeared after a 90-min reaction, assuming that the band signal of the unshifted bands for the Ub2 in each lane at reaction time 0 (zero) was 100%. (**D** and **F**) The percentage of BRAP (124–569), remaining at the end of the reaction (i.e. the non-ubiquitylated BRAP present after a 90-min reaction), was calculated by assessing band signal intensity that corresponded to non-ubiquitylated BRAP (124–569) in each lane after a 90-min reaction, assuming that the band signal for the unshifted Ub2 bands in each lane, at reaction time 0, was 100%. (**E**) To assess total ubiquitylation, the intensity of the high molecular mass signals, detected in the CBB-stained gel after a 90-min reaction, were obtained, and the signals from the same area of the gel at reaction time 0 were subtracted as background. (**G**) To assess the production of auto-ubiquitylated BRAP, the signals, detected in the high molecular mass area of the WB after a 90-min reaction, were obtained, and the signals from the same area of the blot at reaction time 0 were subtracted as background. AU, arbitrary unit of the band signal intensity. These graphs show the average values obtained from two independent experiments.

We next examined whether the RNF52 domain of BRAP exhibits ubiquitin ligase activity against intact ubiquitin chains (oligo-ubiquitin) *in vitro* using all linkage types of Ub2s: K6-Ub2, K11-Ub2, K27-Ub2, K29-Ub2, K33-Ub2, K48-Ub2, K63-Ub2, and M1-Ub2 (Figure 5). We observed that in ubiquitylation, BRAP (124–569) could use all the Ub2 linkage types.

In the reactions using K6-Ub2 and K48-Ub2, which did not bind to BRAP (124–569) in the Biacore assay (Figure 2), the usage of the di-ubiquitin chain (Ub2) (Figure 5C) and signal intensities for total ubiquitylation and auto-ubiquitylated BRAP (Figure 5E,G) were strikingly low, whereas the level of BRAP (124–569) remaining after the 90-min reaction remained high (Figure 5D,F). In the case of K11-Ub2, although the percentage of oligo-Ub usage was low, the signal intensities for total ubiquitylation and auto-ubiquitylation were high, and the level of BRAP (124–569) remaining after the 90-min reaction was low.

In the reactions using K27-Ub2, K29-Ub2, K33-Ub2, K63-Ub2, and M1-Ub2, the usage of oligo-Ub was high and the level of the remaining BRAP (124–569) was low; however, total ubiquitylation and auto-ubiquitylation signal intensities, in the reaction using K27-Ub2, were much lower than those in reactions using K29-Ub2, K33-Ub2, K63-Ub2, and M1-Ub2. Overall, the ubiquitylation reactions using K29-Ub2 and K33-Ub2 proceeded to the greatest extent, and the binding affinity of BRAP (124–569) for K29-Ub2 and K33-Ub2 was lower than that for K63-Ub2 and M1-Ub2.

As described above, Biacore sensorgrams, which correspond to the protein–protein interactions between the RNF52 domain of BRAP (124–569) and these di-ubiquitin chains, did not show significant binding signals for K29 and K33 chains, whereas the K27 chain clearly bound to the RNF52 domain. However, it is difficult to conclude that K29 and K33 chains do not bind to the RNF52 domain because these Biacore sensorgrams, which detect the signals for the binding response, were recognizable in the case of K6, K11, and K48 chains. Therefore, we categorized these protein–protein interaction profiles into the following three groups: the RNF52 domain-binding type [+], non-binding type [−], and faint-binding type [+/−]. K29- and K33-linked chains are categorized into the faint-binding type [+/−], rather than the binding type [+](K27-, K63-, and M1-linked chains) or the non-binding type [−][K6-, K11-, and K48-linked chains (Figure 5C–G, notation at the bottom of each panel)]. Compared with the profiles of protein–protein interactions, the results of the ubiquitylation assay suggest that the recognition ability of the RNF52 domain for the linkage type of the ubiquitin chains is important for the usage of Ub2 (di-ubiquitin chain) but not for the progression of ubiquitylation.

We also examined the ubiquitylation preference of full-length BRAP (1–592) for the different ubiquitin-linkage types and found that full-length BRAP exhibited the same behavior as BRAP (124–569) (Supplementary Data S6A). Therefore, we conclude that the RNF52 domain is responsible for oligomeric ubiquitylation via recognition of ubiquitin-linkage types.

### The RNF52 domain of BRAP can ligate intact M-linked tri- and tetra-ubiquitin chains for ubiquitylation and recognizes structural alteration caused by a phosphomimetic mutation

**Figure 6.**
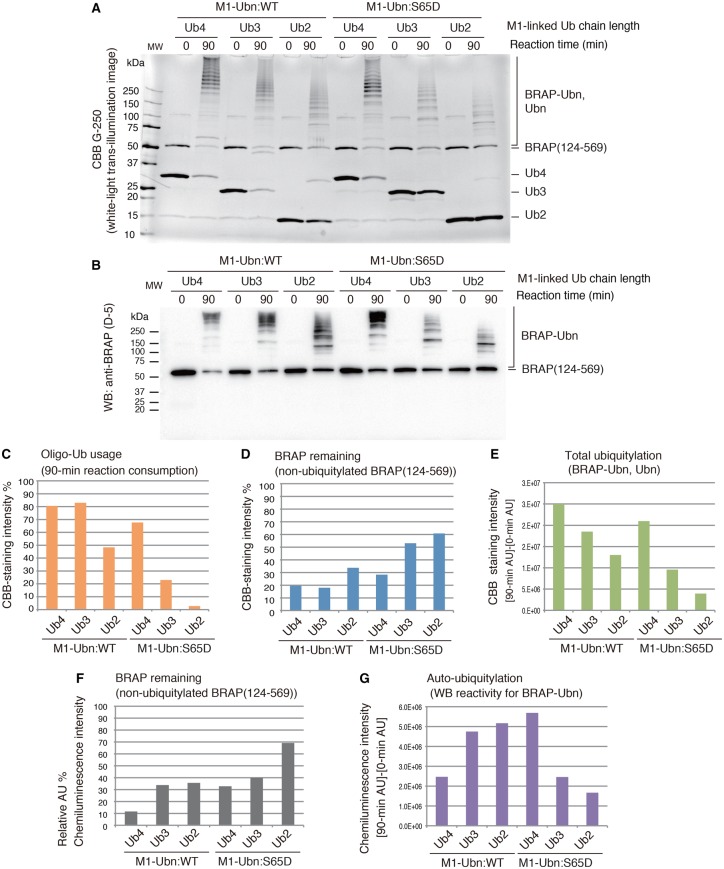
E3 activity of the RNF52 domain of BRAP against M1-linked di-, tri-, and tetra-ubiquitin chains (ubiquitin oligomers) and the phosphomimetic form that consists of S65D ubiquitin mutant. Ubiquitylation assays of the BRAP (124–569) fragment (RNF52 domain of BRAP) in the presence of UBA1, UBE2D1, and M1-linked di-, tri-, and tetra-ubiquitin oligomers (Ub2, Ub3, and Ub4) or their phosphomimetic forms, which consist of the phosphorylation-mimic ubiquitin mutant S65D. (**A**) SDS–PAGE gels of the ubiquitylation reaction products stained with CBB G-250. (**B**) WB of the same samples as in (**A**) probed with an anti-BRAP antibody (clone D-5). (**C**–**G**) The band signals on CBB-stained gels and WB were quantified using the ImageQuant TL software. (**C**) Oligo-Ub usage was calculated by determining what percentage of the band had disappeared after a 90-min reaction, assuming that the band signal of the unshifted bands for the ubiquitin oligomer in each lane at reaction time 0 (zero) was 100%. (**D** and **F**) The percentage of BRAP (124–569) remaining at the end of the reaction (i.e. the non-ubiquitylated BRAP present after a 90-min reaction) was calculated by assessing the band signal intensity that corresponded to non-ubiquitylated BRAP (124–569) in each lane after a 90-min reaction, assuming that the band signal for the unshifted ubiquitin oligomer bands in each lane at reaction time 0 was 100%. (**E**) To assess total ubiquitylation, the intensity of the high molecular mass signals, detected in the CBB-stained gel after a 90-min reaction, was obtained, and the signals from the same area of the gel at reaction time 0 were subtracted as background. (**G**) To assess the production of auto-ubiquitylated BRAP, the signals detected in the high molecular mass area of the WB, after a 90-min reaction, were obtained, and the signals from the same area of the blot at reaction time 0 were subtracted as background. AU, arbitrary unit of the band signal intensity. These graphs show the average values obtained from two independent experiments.

We next examined whether BRAP (124–469) can use M1-linked ubiquitin chains of greater length [i.e. tri-ubiquitin (Ub3) and/or tetra-ubiquitin (Ub4) oligomers] (Figure 6 and Supplementary Data S7). The data indicate that BRAP (124–569) can use these ubiquitin chains for ubiquitylation and auto-ubiquitylation.

We also examined the effect of the ubiquitin phosphomimetic mutation (S65D) on the usage of M1-linked chains and found that BRAP (124–569) can use these phosphomimetic ubiquitin chains (S65D-M1-Ub2, S65D-M1-Ub3, and S65D-M1-Ub3) for ubiquitylation and auto-ubiquitylation (Figure 6). However, the effect of S65D mutation on oligomeric ubiquitylation, using M1-linked ubiquitin chains, differed depending on the length of the ubiquitin chain. In the reactions using S65D-M1-Ub2 and S65D-M1-Ub3, the percentage of oligo-Ub usage and the signal intensities, indicating total ubiquitylation and auto-ubiquitylation, were strikingly decreased compared with reactions using M1-Ub2 and M1-Ub3 (Figure 6C,E,G). In contrast, in the reaction using S65D-M1-Ub4, the oligo-Ub usage and total ubiquitylation were slightly decreased compared with that using M1-Ub4. The quantitative WB analysis revealed that the signal intensity for auto-ubiquitylation in the case of M1-Ub4 was higher than for wild-type Ub4 (Figure 6G). However, the levels of BRAP (124–569), remaining at the end of the 90-min reaction (Figure 6F), detected as non-ubiquitylated BRAP (124–569) using WB with an anti-BRAP antibody, were higher for S65D-M1-Ub4 than for wild-type M1-Ub4; this suggests that the auto-ubiquitylation efficiency of BRAP was lower in the reaction using S65D-M1-Ub4 than that using WT M1-Ub4. We also examined the ubiquitylation preference of full-length BRAP (1–592) for these M1-linked ubiquitin chains (Ub2, Ub3, and Ub4), including the S65D-phosphomimetic form; we found that full-length BRAP exhibited the same behavior as BRAP (124–569) (Supplementary Data S6B).

Using several chain lengths of M1-linked ubiquitin chains (ubiquitin oligomers), including the S65D-phosphomimetic forms, showed that oligomeric ubiquitylation with M1-Ub4 was hardly affected by the phosphomimetic mutation compared with those using M1-Ub2 and M1-Ub3. Therefore, we used a Biacore assay for detailed assessment of protein–protein interactions to examine whether the RNF52 domain of BRAP recognizes differences in these ubiquitin chains.

**Figure 7.**
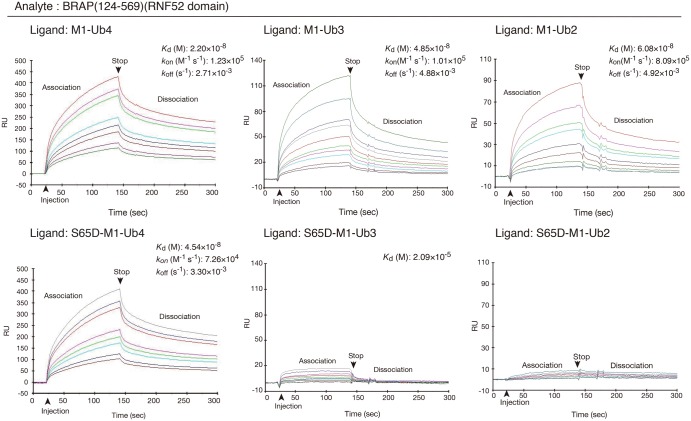
Protein–protein interaction profiles showing binding of the RNF52 domain of BRAP to M1-linked di-, tri-, and tetra-ubiquitin chains (ubiquitin oligomers). Surface plasmon resonance sensorgrams from the Biacore assay (i.e. protein–protein interaction profiles) are shown. Resonance signal, expressed as response unit (RU), shows the degree of the binding of BRAP to the ubiquitin oligomers. The immobilized ligands [i.e. M1-linked di-, tri-, and tetra-ubiquitin oligomers (Ub2, Ub3, and Ub4), and their phosphomimetic forms that consisted of the S65D mutation] are shown in the top left of each panel. The analyte (applied as a mobile molecule to an immobilized ligand on the sensor chip) was BRAP (124–569) (RNF52 domain). The measured equilibrium dissociation constant (*K*_d_), association rate constant (*k*_on_), and dissociation rate constant (*k*_off_), for each binding, are shown at the top right of each panel where possible.

The Biacore sensorgrams for the interaction between BRAP (124–569) and M1-Ub4, or the interaction between BRAP (124–569) and the S65D-M1-Ub4 mutant, differed from the ones for BRAP (124–569) binding to M1-Ub2/S65-M1-Ub2 and M1-Ub3/S65-M1-Ub3 (Figure 7). The binding affinities of BRAP (124–569) for M1-Ub2 and M1-Ub3 were significantly affected by the S65D-phosphomimetic mutation. In contrast, the binding of BRAP (124–569) to M1-Ub4 was not significantly affected by the phosphomimetic S65D ubiquitin mutation. Therefore, we conclude that the effect of phosphomimetic S65D ubiquitin mutation on the ligation of M1-Ub4 differed from that on the ligation of M1-Ub3 and M1-Ub2.

Based on these experiments using BRAP (124–569) and M1-linked ubiquitin chains of various lengths (Ub2, Ub3, and Ub4), we conclude that the RNF52 domain of BRAP recognizes the structural differences between these modified ubiquitin chains before it ligates them.

### The types of oligomeric ubiquitin-chain linkages catalyzed by the RNF52 domain of BRAP

**Figure 8.**
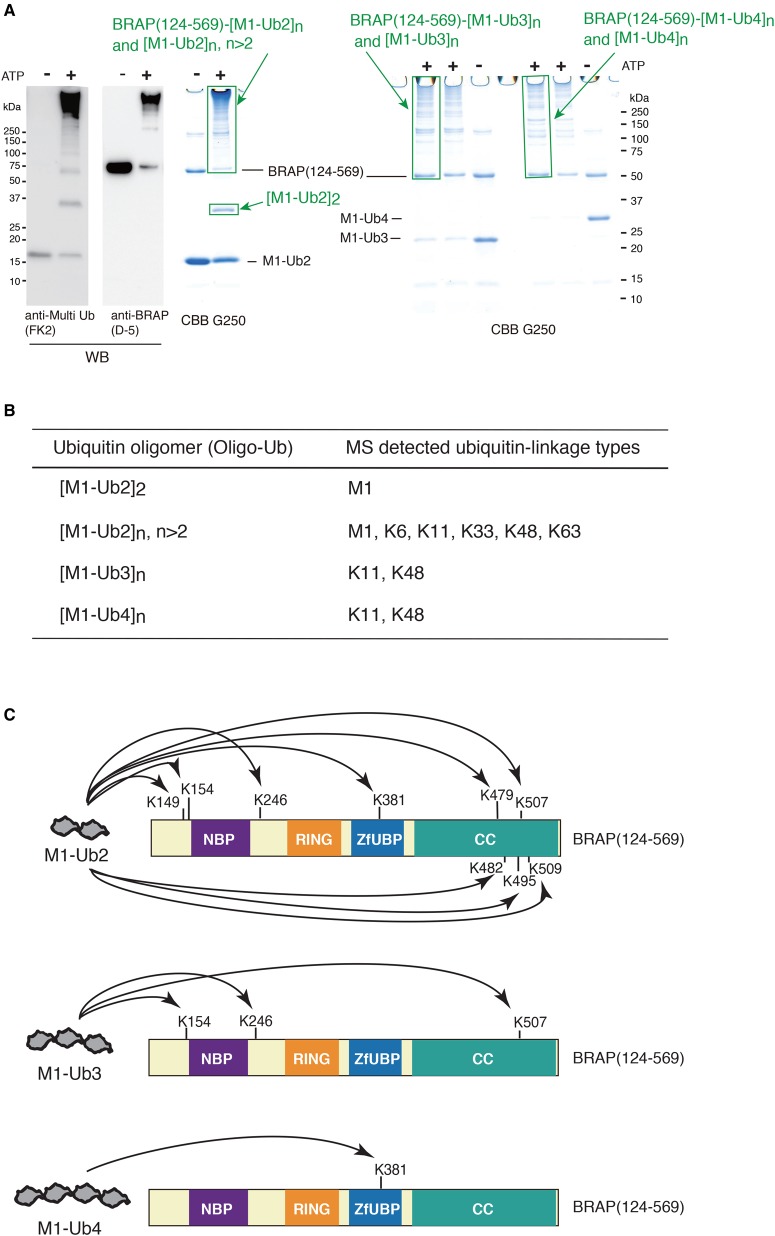
Summary of mass spectrometry analysis of the products from BRAP (124–569) (RNF52 domain)-mediated *in vitro* ubiquitylation using M1-linked ubiquitin oligomers. (**A**) Images of SDS–PAGE/CBB-stained gels, showing the gel slices used for mass spectrometry analysis, are highlighted using green boxes and labeled with a green arrow. These oligomeric ubiquitylation samples were taken after a 90-min reaction at 37°C, with or without ATP. The two left-hand side panels are WBs of the M1-Ub2 oligomeric ubiquitylation probed with either an anti-multi Ub (FK2) antibody or an anti-BRAP (D-5) antibody. (**B**) Variation in ubiquitin-chain-linkage types produced by the RNF52 domain [BRAP (124–569)]-mediated oligomeric ubiquitylation. (**C**) Schematic showing the effect of ubiquitin-chain length on auto-ubiquitylation. Mass spectra and details of the mass spectrometry analysis are provided in Supplementary Data S8 and S9.

We employed MS to analyze the reaction products of the oligomeric ubiquitylation using Ub2, Ub3, and Ub4, in order to explore ubiquitin-linkage types and auto-ubiquitylated sites on the RNF52 domain. A summary of the results is shown in Figure 8; mass spectra and details are provided in Supplementary Data S8 and S9.

Peptide mass spectrometry, conducted on a gel slice obtained from a high molecular mass area of SDS–PAGE (Figure 8A), detected several types of K-linkages; however, the types of linkage differed depending on the type of ubiquitin oligomer (Figure 8B and Supplementary Data S8). M1, K6, K11, K33, K48, and K63 were detected in the sample [M1-Ub2]*n* (*n* > 2) (Supplementary Data S8A), whereas K11 and K48 were detected in samples [M1-Ub3]*n* and [M1-Ub4]*n* (Supplementary Data S8B,C).

In the SDS–PAGE analysis of the reaction products derived using M1-Ub2, we observed extra bands, indicating that two Ub2s had become connected (Figure 8A: [M1-Ub2]2); this was detected by CBB staining and WB analysis with an anti-multi-ubiquitin antibody. MS analysis of the peptides, from the sample corresponding to the [M1-Ub2]2 band, did not indicate any K-linkages (Supplementary Data S8D), suggesting that an M1-linked oligomeric chain was generated in this reaction.

Moreover, MS analysis of auto-ubiquitylated sites, produced during the oligomeric ubiquitylation reaction with M1-Ub2, -Ub3, and -Ub4, revealed that these auto-ubiquitylated sites differ from each other (Figure 8C and Supplementary Data S9): K149, K154, K246, K381, K479, K482, K495, K507, and K509 were detected in sample BRAP (124–569)–[M1-Ub2]*n* (Supplementary Data S9A1 and SA2); K154, K246, and K507 were detected in sample BRAP (124–569)–[M1-Ub3]*n* (Supplementary Data S9B); and K381 was detected in sample BRAP(124–569)–[M1-Ub4]*n* (Supplementary Data S9C).

These results indicate that chain length enables the RNF52 domain of BRAP to recognize structural variation in ubiquitin chains before it ligates them.

## Discussion

In the present study, we determined the underlying characteristics of the RNF52 domain of BRAP [BRAP (124–569)]. The RNF52 domain preferentially binds to ubiquitin chains (oligo-ubiquitin), but not to the ubiquitin monomer. The RNF52 domain can utilize not only ubiquitin monomers, but also ubiquitin chains for ubiquitylation. Interestingly, use of oligo-ubiquitin was significantly higher than that of mono-ubiquitin in the RNF52 domain catalyzed ubiquitylation (Figures 4 and 5). Furthermore, we found that the RNF52 domain of BRAP catalyzes ubiquitylation using various ubiquitin chains (oligo-ubiquitin) by recognizing the ubiquitin-chain types. Additionally, the RNF52 domain recognizes the structure of M1-linked ubiquitin chains, including the ubiquitin-linkage type and structural alterations caused by chain length and mutation of a kinase site, to produce a phosphomimetic (S65D).

However, the molecular mechanism enabling this oligomeric ubiquitylation remained unclear. A recent crystal structural analysis of M1-linked chains demonstrated that ABIN-2, which belongs to the UBAN family (Figure 1D), recognizes M1-linked tri-ubiquitin (Ub3), and M1-linked Ub3 can form a right-handed helical trimer to bridge the two proteins [38]. Additionally, phosphorylated ubiquitin oligomers reportedly undergo structural changes [35]. Our experiments showed that the RNF52 domain can recognize structural alterations of M1-linked ubiquitin chains caused by chain length and phosphomimetic mutations (Figures 6–8). Therefore, the modified ubiquitin oligomer structure-sensing function of the RNF52 domain is required for oligomeric ubiquitylation. To determine the molecular mechanism of oligomeric ubiquitin-linked extra-chain elongation, it will be necessary to determine the crystal structures of the protein complexes consisting of the ubiquitin oligomer-charged E2 and ubiquitin oligomer-holding RNF52 domains. One of the features of the RNF52 domain architecture is that contains an NBP as a subdomain. Based on the crystal structure of homoserine kinase and ATP complex, an α/β plait fold that participates in nucleotide-binding was identified in a previous study [39]. Therefore, the NBP subdomain of the RNF52 domain, which also contains an α/β plait, may contribute to oligomeric ligation by recruiting ATP along with E1; however, this hypothesis has not been confirmed. We expect that this molecular mechanism will be revealed in future structural analyses.

**Figure 9.**
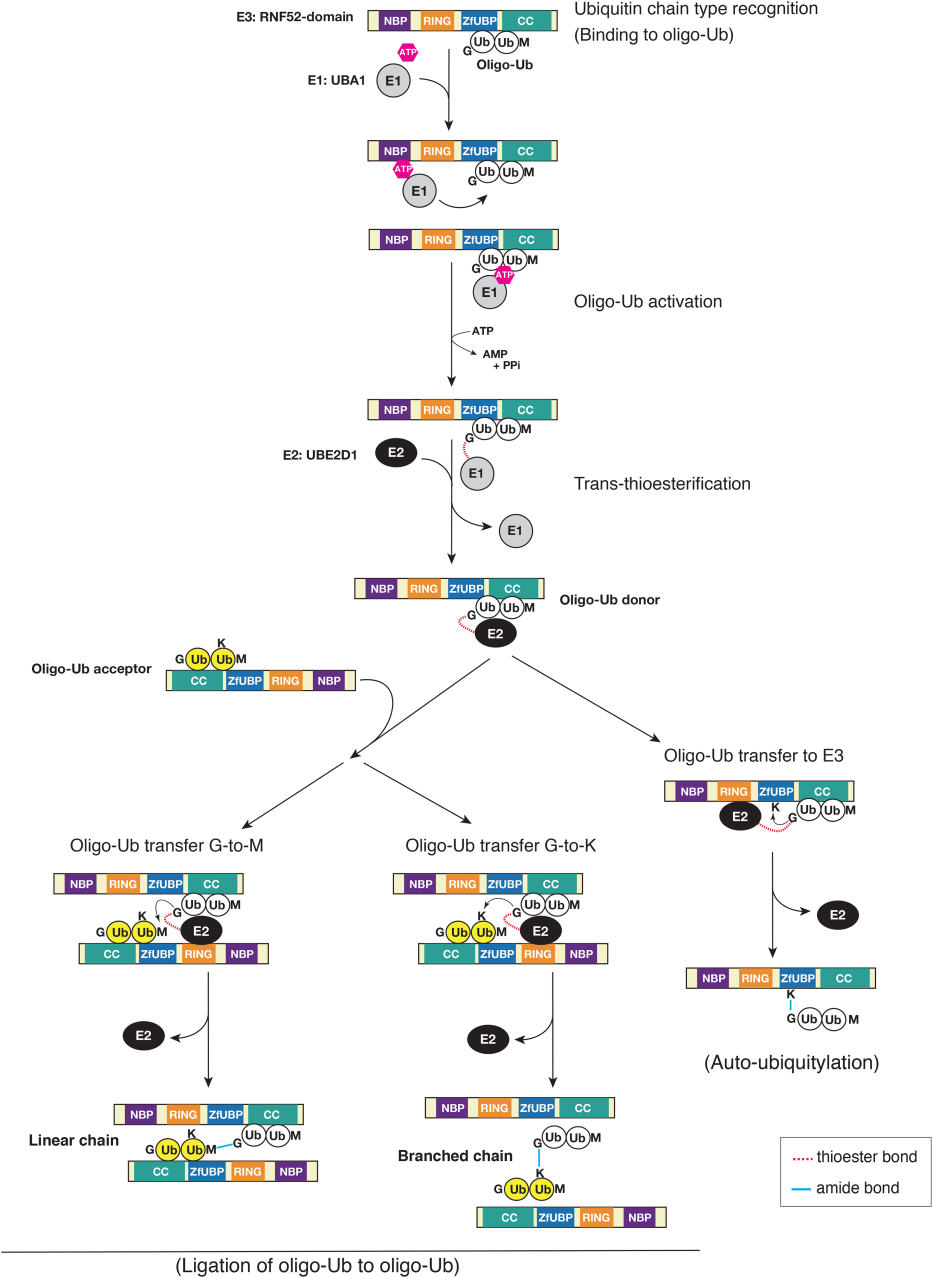
Diagrammatic representation of oligomeric ubiquitylation by the central catalytic domain (RNF52 domain) of BRAP using M1-linked ubiquitin oligomers (oligo-Ub). A proposed model for the mechanism of oligomeric ubiquitylation catalyzed by the RNF52 domain of BRAP with UBE2D1 (E2) and UBA1 (E1). Details are described in the Discussion section.

Here, we propose a model for the mechanism of ubiquitylation, utilizing oligo-ubiquitin (referred to as ‘oligomeric ubiquitylation’ in the present study), controlled by the RNF52 domain of BRAP (Figure 9). In this model, the RNF52 domain (the central catalytic domain of BRAP) recognizes and binds to both the acceptor and donor ubiquitin chains (oligo-Ub in Figure 9). This is the first step in oligomeric ubiquitylation. In the second step, oligo-Ub, bound to the RNF52 domain, recruits ATP along with E1- or the ATP-binding E1 via the NBP subdomain, where E1 activates oligo-Ub on the RNF52 domain using ATP; hence, the C-terminal glycine (G) of oligo-Ub is adenylated. Simultaneously, E1 recruits E2 and the activated ubiquitin is transferred to E2 in a *trans*-thioesterification reaction. While the E2-Ub complex is tethered transiently to the RING subdomain, E2 is released from the RNF52 domain. UBE2D1 has weak binding affinity for the RNF52 domain (Supplementary Data S4B); the RNF52 domain holds the oligo-Ub in place. In contrast, the RNF52 domain, bound to the donor oligo-Ub, recruits another RNF52 domain, which holds the acceptor oligo-Ub; these are then combined and form a ubiquitin link via covalent attachment of the C-terminal G from donor oligo-Ub to M (N-terminal methionine) or K (internal lysine) of the acceptor oligo-Ub. In our study, the RNF52 domain bound preferentially to M1-Ub2 and K63-Ub2 (Figure 2), but not to K6-, K11-, or K48-Ub2. Therefore, if a K6/K11/K48-linked chain with a K–G linkage is generated, the newly synthesized oligo-Ub will dissociate from the catalytic region. For auto-ubiquitylation, the activated oligo-Ub C-terminal G will be transferred to K on the RNF52 domain, which holds the oligo-Ub directly, while releasing both E1 and E2 from the catalytic region.

In the ubiquitin system, the conventional model for the mechanism of ubiquitin-chain assembly indicates that ubiquitin-chain elongation occurs one-by-one [3,7]. Sequential transfer of single ubiquitin molecules by a quantitative framework, based on product distribution, predicts that SCF complex E3s (such as SCF Cdc4 and SCF β-TrCP) co-operate with an E2 (Cdc34) to build polyubiquitin chains on the substrates [40]. However, it is possible that the synthesis of ubiquitin chains uses di-ubiquitin chains as building blocks [22]. A previous study demonstrated that E2 (Ube2g2)-mediated polyubiquitylation involves preassembly of K48-linked chains at the E2 catalytic cysteine and that polyubiquitylation of a substrate can be achieved by transferring preassembled ubiquitin chains from E2 to a lysine residue on the substrate [41]. Our results also indicate that ubiquitylation is not always a sequential assembly process involving the transfer of individual ubiquitin molecules.

The present study did not examine the existence of oligomeric ubiquitylation *in vivo* M1-linked ubiquitin oligomer connected via K6, K11, K33, K48, or K63 linkages, or the biological function of oligomeric-ligated ubiquitin complexes generated by the combination of the RNF52 domain of BRAP and UBE2D1. From a biological perspective, BRAP was reported to be involved in the inhibitor-κB kinase/NF-κB signaling cascade [13,15,42]. BRAP also interacts with galectin-2 [13], which can interact with NEMO [43]. It has been reported that M1-linked linear ubiquitin interacts with NEMO and modulates activation of NF-κB [44,45]. In addition, it has recently been reported that K48–K63 branched ubiquitin linkages are abundant in cells and regulate NF-κB signaling [46]. Based on these studies and our findings that the RNF52 domain of BRAP binds and has E3 activity against M1-linked ubiquitin oligomers, the E2–E3 combination of UBE2D1 and RNF52 domain catalyzes ubiquitin-chain branching from the M1-linked chain to K48- and K63-linked chain. Thus, oligomeric-ligated ubiquitin complexes generated by the RNF52 domain may provide a modified platform for NF-κB signal transduction.

The existence and subcellular localization of ubiquitin system components, including E2, E3, pools of cellular ubiquitin, and ubiquitin chains, are very diverse in cells and tissues [47,48]. In our ubiquitylation assays, UBE2D1 was used as the E2 partner of BRAP; however, examination of other members of the UBE2D family as the E2 partner may reveal other function(s). In summary, we observed the preferential E2 usage by BRAP and specific ubiquitin-linkage types catalyzed by the RNF52 domain of BRAP. The domain architecture of the RNF52 domain, which consists of four subdomains, NBP–RING–ZfUBP–CC, is conserved in eukaryotic orthologs. We expect that characterization of the RNF52 domain will be useful for further understanding the molecular mechanisms underlying human diseases and eukaryotic cellular signaling.

## Abbreviations

ABIN: human A20-associating cytosolic
ATP: adenosine triphosphate
BRAP: BRCA1-associated protein 2
BRCA1: breast cancer type 1 susceptibility protein
CBB: Coomassie Brilliant Blue
CC: coiled-coil
DTT: dithiothreitol
HEPES: 4-(2-hydroxyethyl)-1-piperazineethanesulfonic acid
HGNC: human genome organization (HUGO) Nomenclature Committee
MAPK: mitogen-activated protein kinase
MBP: maltose-binding protein
MS: mass spectrometry
NBP: nucleotide-binding α/β plait
NEMO: NF-κB essential modulator
NF-κB: nuclear factor kappa-light-chain-enhancer of activated B cells
oligo-Ub: ubiquitin oligomer
RING: really interesting new gene
RNF52: RING-finger protein 52
SCF complex: Skp–Cullin-F-box containing complex
SDS–PAGE: sodium dodecyl sulfate–polyacrylamide electrophoresis
Skp: S-phase kinase-associated protein
SPR: surface plasmon resonance
TEV: Tobacco Etch virus
UBA1: ubiquitin-activating enzyme E1
UBE2: ubiquitin-conjugating enzyme E2
UBP: ubiquitin-specific protease
UniProtKB: UniProt Knowledgebase
WB: Western blot
WT: wild type
ZfUBP: UBP-like zinc finger

## Supplementary Material

Supplementary Datas
