# Supplementary material for: Central catalytic domain of BRAP (RNF52) recognizes the types of ubiquitin chains and utilizes oligo-ubiquitin for ubiquitylation
Source: Biochem J. 2017 Sep 8;474(18):3207–26. doi: 10.1042/BCJ20161104 (PMC5628404; doi:10.1042/BCJ20161104)
Supplement: Supplementary Datas [file BCJ-474-3207-s1.pdf]

BCJ20161104

Supplementary material

Central catalytic domain of BRAP (RNF52) recognizes the types of ubiquitin chains and utilizes oligo-ubiquitin for ubiquitylation

Shisako Shoji[1], Kazuharu Hanada [1], Noboru Ohsawa[1], and Mikako Shirouzu [1, 2]

[1]Division of Structural and Synthetic Biology, RIKEN Center for Life Science Technologies, 1-7-22 Suehiro-cho, Tsurumi-ku, Yokohama 230-0045, Japan; [2]Program for Drug Discovery and Medical Technology Platforms; RIKEN, 1-7-22 Suehiro-cho, Tsurumi-ku, Yokohama 230-0045, Japan

Correspondence: Shisako Shoji (shisako.shoji@riken.jp) or Mikako Shirouzu (mikako.shirouzu@riken.jp)

Supplementary data captions and legends

Supplementary Data S1. Multiple sequence alignment for RNF52 proteins from representative eukaryotes

Multiple sequence alignment was performed using the Clustal Omega program (http://www.ebi.ac.uk/Tools/msa/clustalo/). Amino-acid sequences were obtained from the UniProt Knowledgebase (UniProtKB).

Supplementary Data S2. SDS-PAGE/CBB-stained gels of the purified protein used in the Biacore experiments

(*) N11-tag M1-Ub2 (M1-linked di-ubiquitin N-terminally fused with a modified natural poly-histidine N11-tag) was not used in this study.

Supplementary Data S3. E3 activity of BRAP(1-592) in the presence of various E2s, and Biacore analysis of the binding of BRAP(1-592) and BRAP(124-569) to UBE2D1

(**A**) The E3 activity of BRAP in the presence of various E2s. Representative SDS-PAGE gels showing the *in vitro* ubiquitylation profiles obtained using an E2 scan plate (Ubiquigent E2 scan Kit, version 2). Reactions were performed in the presence of ATP, UBA1(E1), ubiquitin, and the indicated E2 proteins and wild-type BRAP(1-592) or its RING-finger mutant (C264S). The top panels show western blot (WB) analysis of the *in vitro* reaction products using an anti-multi ubiquitin antibody (clone FK2). The FK2 antibody recognizes ubiquitin conjugates, but not free ubiquitin. The bottom panels show the SDS-PAGE gels of the *in vitro* reaction products stained with Coomassie brilliant blue (CBB) G-250. (**B**) Surface plasmon resonance sensorgrams showing the protein-protein interaction assay, using the Biacore system, for the interaction between UBE2D1 and BRAP(1-592) (full-length) or BRAP(124-569) (RNF52-domain).

Supplementary Data S4. The E3 activity of BRAP (124-569) on various ubiquitin mutants

Ubiquitylation assays of the BRAP(124–569) fragment (RNF52-domain of BRAP) in the presence of UBA1(E1), UBE2D1, and wild-type ubiquitin (WT) or the ubiquitin mutants S65D, K6R, K11R, K27R, K29R, K33R, K48R, K63R, Me (methylated ubiquitin), K0 (no lysine residue), K6 only, or K63 only. SDS-PAGE gels of the *in vitro* reaction products, stained with CBB G-250, are shown on the left-hand side. The right-hand side shows a WB analysis of the *in vitro* reaction products using an anti-BRAP antibody (clone D-5) and an anti-multi-ubiquitin antibody (clone FK2), which recognizes both mono- and poly-ubiquitylated species but not free ubiquitin. The data shown in the top and bottom panels are from experiments performed independently of each other.

Supplementary Data S5. Mass spectrometry data for the ubiquitylation products using the E2•E3 combination UBE2D1•BRAP (124-569) (RNF52-domain) with ubiquitin monomer (mono-ubiquitin, Ub1)

(**A**) Images of SDS-PAGE/CBB stained gels, showing the gel slices used for mass spectrometry analysis, are highlighted using green boxes and labeled with a green arrow. These ubiquitylation samples were taken after a 90-min reaction at 37°C, with or without ATP. (**B**) A western blot (WB) of the ubiquitylation probed with an anti-multi Ub (FK2) antibody. (**C1–2**) Mass spectra of ubiquitin-linkage types generated in monomeric ubiquitylation.

Supplementary Data S6. Oligomeric ubiquitylation by full-length BRAP

Ubiquitylation assays assessing full-length BRAP [BRAP(1–592)] in the presence of UBA1(E1), UBE2D1, and various ubiquitin oligomers. SDS–PAGE gels stained with CBB G-250 and a western blot (WB) of the ubiquitylation reaction products using an anti-BRAP antibody (clone D-5). (**A**) The E3 activity of BRAP(1-592) against all linkage types of all linkage types of Ub2s. (**B**) E3 activity of BRAP(1-592) against M1-linked Ub2, Ub3, and Ub4 chains and against phosphomimetic forms containing the S65D ubiquitin mutation.

Supplementary Data S7. Images of the SDS-PAGE gel using samples from ubiquitylation reactions with Ub1, Ub2, Ub3, Ub4, and RING-finger mutant (C264S) of the RNF52-domain, BRAP(124-569)

The top panel shows SDS-PAGE gels, stained with CBB G-250, of the *in vitro* reaction products. The bottom panel shows a western blot (WB) of the *in vitro* reaction products using an anti-BRAP antibody (clone D-5). Ubiquitylation reactions using a RING-finger mutant (C264S) of BRAP(124-569) after a longer incubation time (90 min $\rightarrow$ 180 min); monomeric ubiquitylation was detected in the case of Ub1, whereas oligomeric ubiquitylation was not detected in the case of Ub2, Ub3, and Ub4. Monomeric ubiquitylation was likely catalyzed by UBE2D1, which can catalyze K11- and K48-linkages in the absence of E3.

Supplementary Data S8. Mass spectra of ubiquitin-linkage types, generated in oligomeric ubiquitylation, using the E2•E3 combination of the combined UBE2D1•BRAP(124-569) (RNF52-domain)

Supplementary Data S9. Mass spectra of auto-ubiquitylated sites of BRAP(124-569) (RNF52-domain), generated in oligomeric ubiquitylation, using the E2•E3 combination of UBE2D1•BRAP(124-569) (RNF52-domain)

BCJ20161104_**Supplementary Data S1**

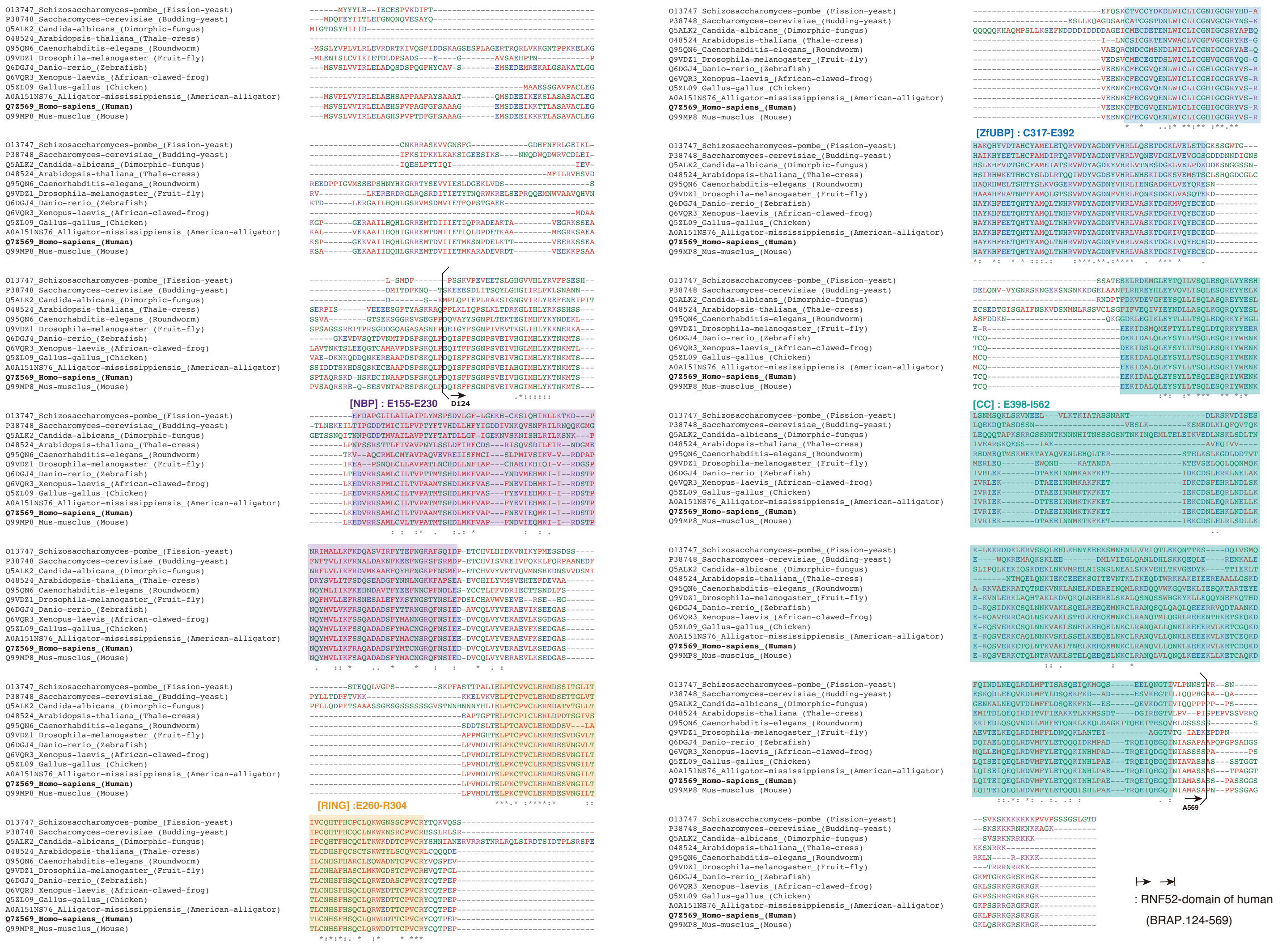

BCJ20161104_**Supplementary Data S2**

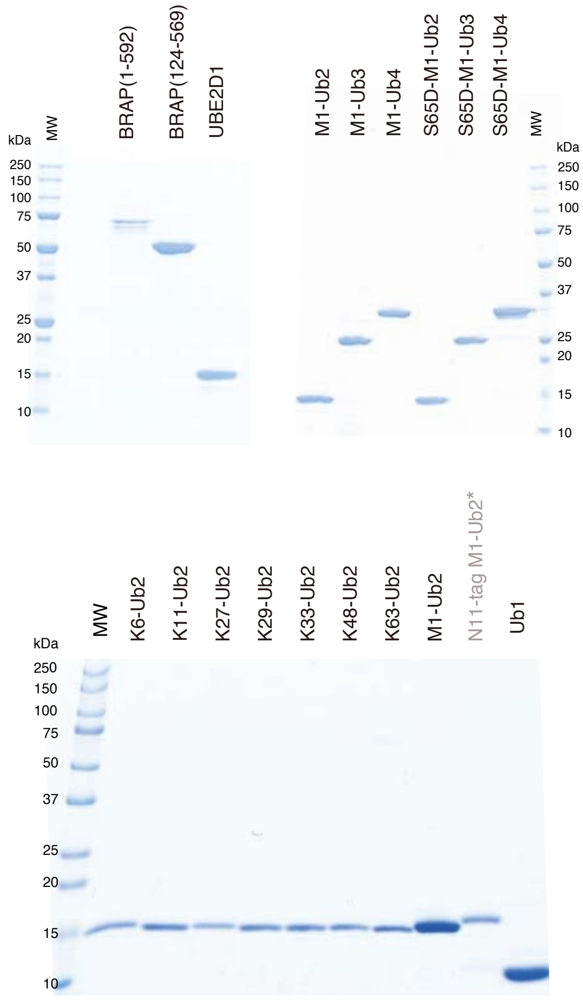

SDS-PAGE/CBB G-250 staining

A

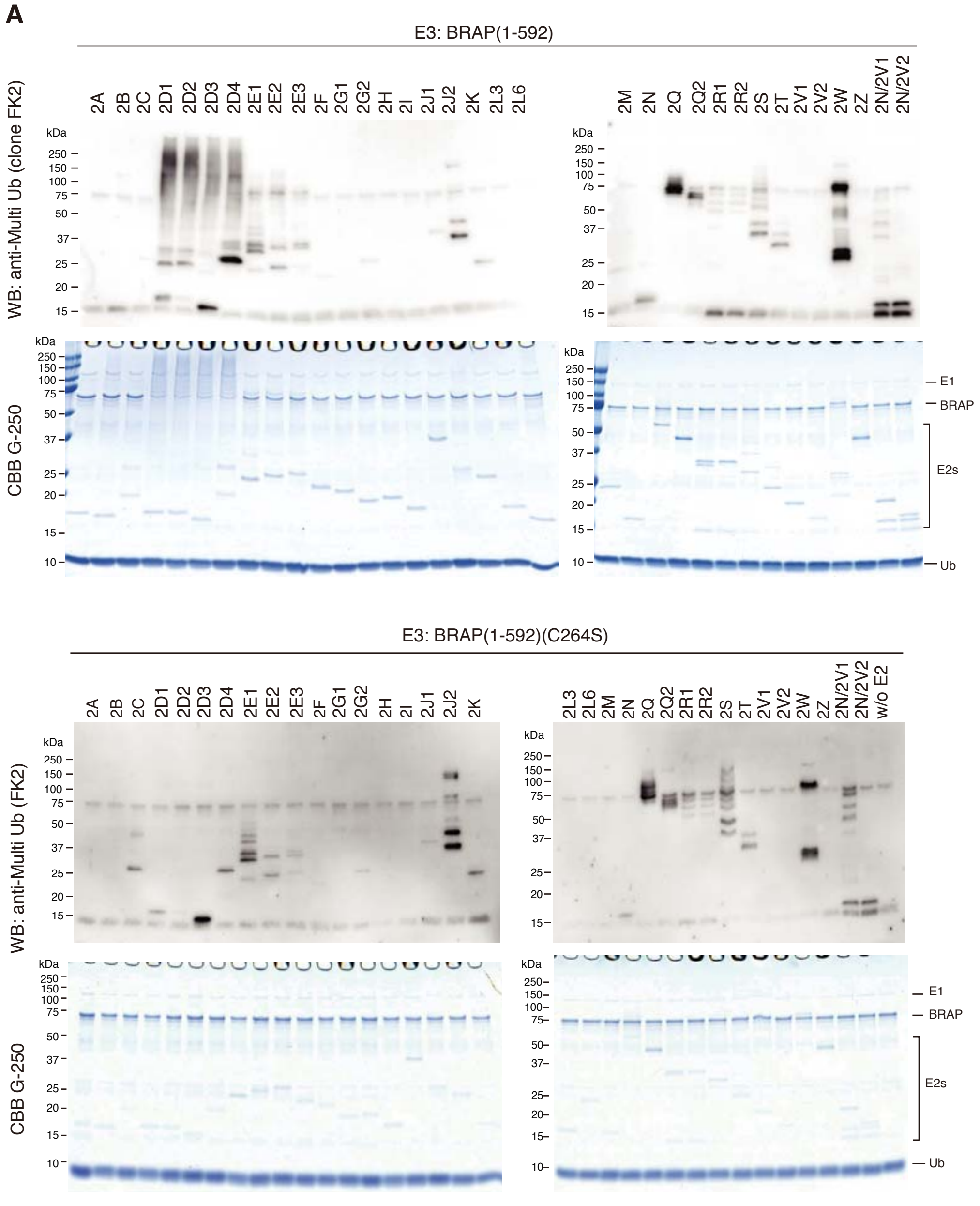
E3: BRAP(1-592)
WB: anti-Multi Ub (clone FK2)
2A 2B 2C 2D1 2D2 2D3 2D4 2E1 2E2 2E3 2F 2G1 2G2 2H 2I 2J1 2J2 2K 2L3 2L6
2M 2N 2Q 2Q2 2R1 2R2 2S 2T 2V1 2V2 2W 2Z 2N/2V1 2N/2V2
kDa
250 150 100 75 50 37 25 20 15 10
CBB G-250
E1
BRAP
E2s
Ub
E3: BRAP(1-592)(C264S)
WB: anti-Multi Ub (FK2)
2A 2B 2C 2D1 2D2 2D3 2D4 2E1 2E2 2E3 2F 2G1 2G2 2H 2I 2J1 2J2 2K
2L3 2L6 2M 2N 2Q 2Q2 2R1 2R2 2S 2T 2V1 2V2 2W 2Z 2N/2V1 2N/2V2 w/o E2
CBB G-250
E1
BRAP
E2s
Ub

B

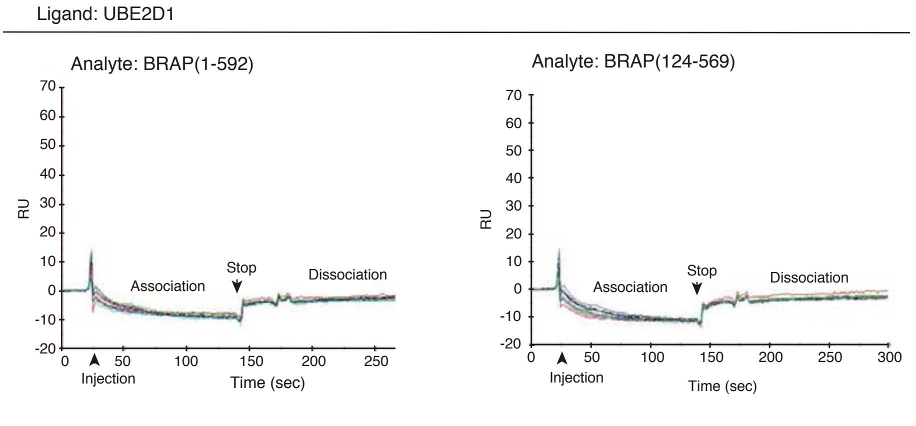
Ligand: UBE2D1
Analyte: BRAP(1-592)
Analyte: BRAP(124-569)
RU
70 60 50 40 30 20 10 0 -10 -20
Association
Stop
Dissociation
Injection
0 50 100 150 200 250 300
Time (sec)

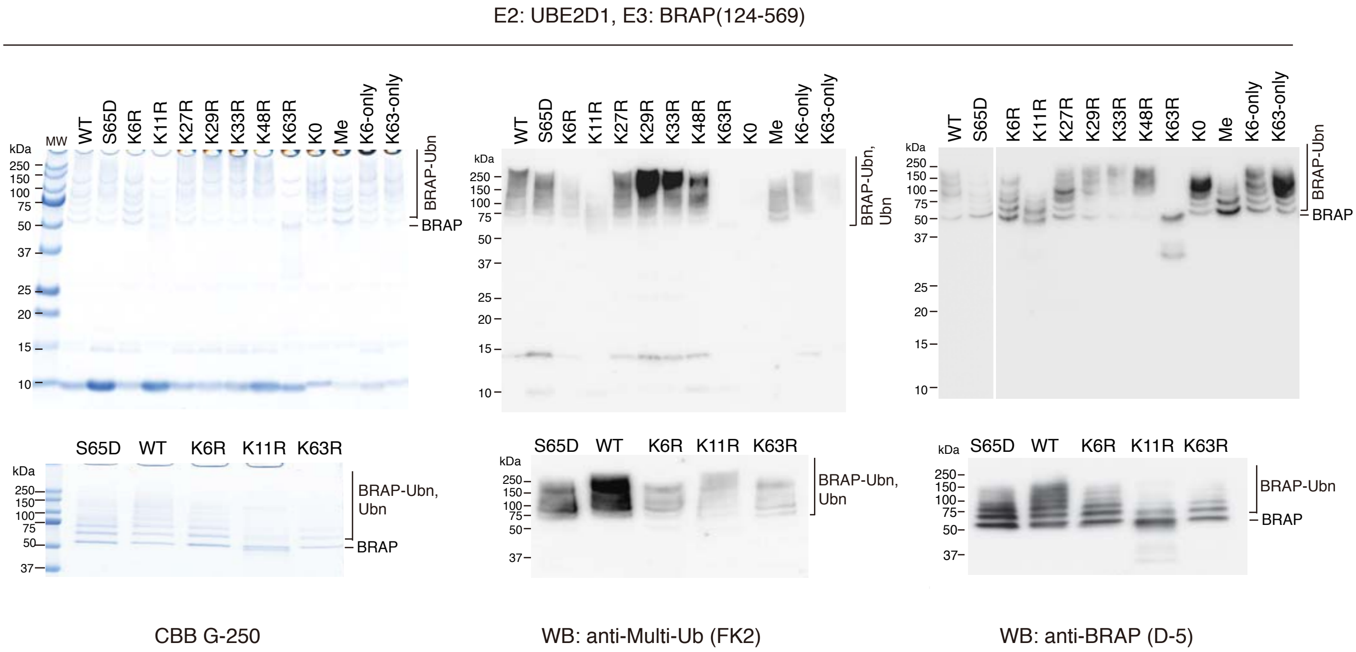
E2: UBE2D1, E3: BRAP(124-569)
MW
WT
S65D
K6R
K11R
K27R
K29R
K33R
K48R
K63R
K0
Me
K6-only
K63-only
kDa
250
150
100
75
50
37
25
20
15
10
BRAP-Ubn
BRAP
BRAP-Ubn, Ubn
CBB G-250
WB: anti-Multi-Ub (FK2)
WB: anti-BRAP (D-5)

A

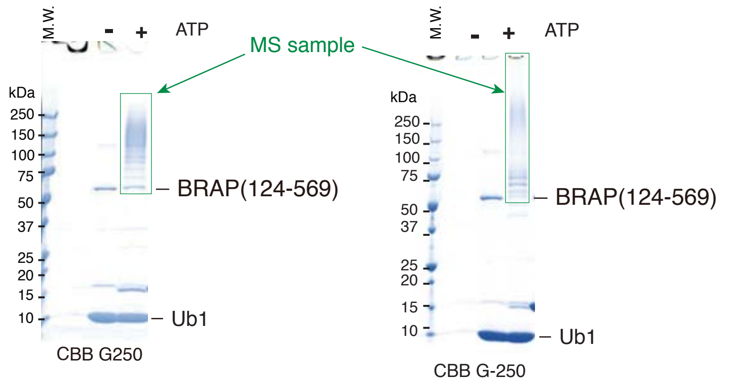

B

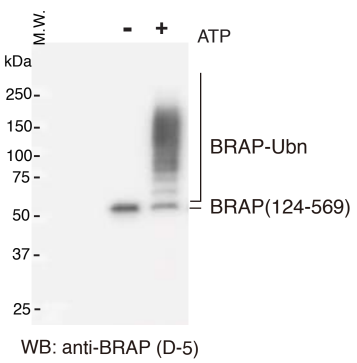

C1

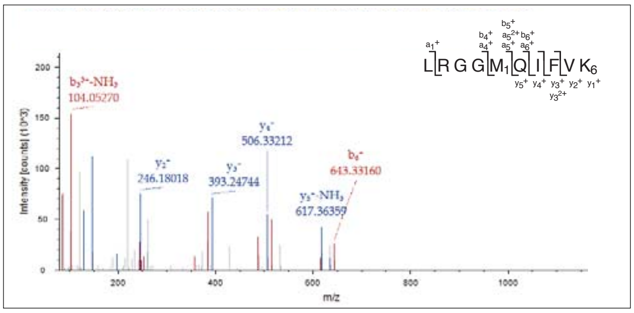

Peptide Summary

Sequence: LRGGMQIFVK, Charge: +3, Monoisotopic m/z: 383.55844 Da (-0.05 mmu/-0.14 ppm), MH+: 1148.66077 Da, RT: 45.40 min,
Identified with: Mascot (v1.30); IonScore:21, Exp Value:8.5E-002, Ions matched by search engine: 9/104
Fragment match tolerance used for search: 20 mmu
Fragments used for search: a; a-NH_3; b; b-NH_3; y; y-NH_3

Fragment Matches

Value Type: Theo. Mass [Da]

Ion Series | Neutral Losses | Precursor Ions

#1	a+	a2+	a3+	b+	b2+	b3+	Seq.	y+	y2+	y3+	#2
1	86.09643	43.55185	29.37033	114.09135	57.54931	38.70197	L				10
2	242.19755	121.60241	81.40404	270.19247	135.59987	90.73567	R	1035.57686	518.29207	345.86380	9
3	299.21902	150.11315	100.41119	327.21394	164.11061	109.74283	G	879.47574	440.24151	293.83010	8
4	356.24049	178.62388	119.41835	384.23541	192.62134	128.74999	G	822.45427	411.73077	274.82294	7
5	487.28099	244.14413	163.09852	515.27591	258.14159	172.43015	M	765.43280	383.22004	255.81578	6
6	615.33957	308.17342	205.78471	643.33449	322.17088	215.11635	Q	634.39230	317.69979	212.13562	5
7	728.42364	364.71546	243.47940	756.41856	378.71292	252.81104	I	506.33372	253.67050	169.44942	4
8	875.49206	438.24967	292.50221	903.48698	452.24713	301.83384	F	393.24965	197.12846	131.75473	3
9	974.56048	487.78388	325.52501	1002.55540	501.78134	334.85665	V	246.18123	123.59425	82.73193	2
10							K	147.11281	74.06004	49.70912	1

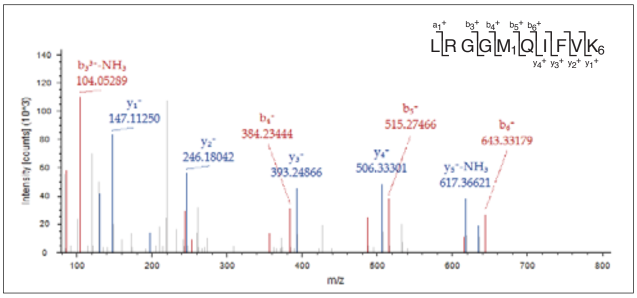

Peptide Summary

Sequence: LRGGMQIFVK, Charge: +3, Monoisotopic m/z: 383.55884 Da (+0.34 mmu/+0.9 ppm), MH+: 1148.66196 Da, RT: 45.48 min,
Identified with: Mascot (v1.30); IonScore:25, Exp Value:3.5E-002, Ions matched by search engine: 5/104
Fragment match tolerance used for search: 20 mmu
Fragments used for search: a; a-NH_3; b; b-NH_3; y; y-NH_3

Fragment Matches

Value Type: Theo. Mass [Da]

Ion Series | Neutral Losses | Precursor Ions

#1	a+	a2+	a3+	b+	b2+	b3+	Seq.	y+	y2+	y3+	#2
1	86.09643	43.55185	29.37033	114.09135	57.54931	38.70197	L				10
2	242.19755	121.60241	81.40404	270.19247	135.59987	90.73567	R	1035.57686	518.29207	345.86380	9
3	299.21902	150.11315	100.41119	327.21394	164.11061	109.74283	G	879.47574	440.24151	293.83010	8
4	356.24049	178.62388	119.41835	384.23541	192.62134	128.74999	G	822.45427	411.73077	274.82294	7
5	487.28099	244.14413	163.09852	515.27591	258.14159	172.43015	M	765.43280	383.22004	255.81578	6
6	615.33957	308.17342	205.78471	643.33449	322.17088	215.11635	Q	634.39230	317.69979	212.13562	5
7	728.42364	364.71546	243.47940	756.41856	378.71292	252.81104	I	506.33372	253.67050	169.44942	4
8	875.49206	438.24967	292.50221	903.48698	452.24713	301.83384	F	393.24965	197.12846	131.75473	3
9	974.56048	487.78388	325.52501	1002.55540	501.78134	334.85665	V	246.18123	123.59425	82.73193	2
10							K	147.11281	74.06004	49.70912	1

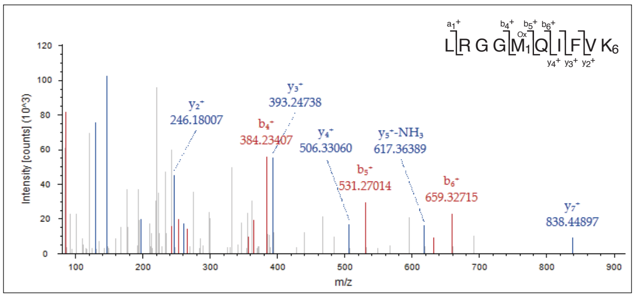

Peptide Summary

Sequence: LRGGMQIFVK, M5-Oxidation (15.99492 Da)
Charge: +3, Monoisotopic m/z: 388.89020 Da (+0.06 mmu/+0.17 ppm), MH+: 1164.65604 Da, RT: 34.32 min,
Identified with: Mascot (v1.30); IonScore:33, Exp Value:4.6E-003, Ions matched by search engine: 8/104
Fragment match tolerance used for search: 20 mmu

Fragment Matches

Value Type: Theo. Mass [Da]

Ion Series | Neutral Losses | Precursor Ions

#1	a+	a2+	a3+	b+	b2+	b3+	Seq.	y+	y2+	y3+	#2
1	86.09643	43.55185	29.37033	114.09135	57.54931	38.70197	L				10
2	242.19755	121.60241	81.40404	270.19247	135.59987	90.73567	R	1051.57178	526.28953	351.19544	9
3	299.21902	150.11315	100.41119	327.21394	164.11061	109.74283	G	895.47066	448.23897	299.16174	8
4	356.24049	178.62388	119.41835	384.23541	192.62134	128.74999	G	838.44919	419.72823	280.15458	7
5	503.27591	252.14159	168.43015	531.27082	266.13905	177.76179	M-Oxidation	781.42772	391.21750	261.14742	6
6	631.33449	316.17088	211.11635	659.32940	330.16834	220.44798	Q	634.39230	317.69979	212.13562	5
7	744.41856	372.71292	248.81104	772.41347	386.71037	258.14267	I	506.33372	253.67050	169.44942	4
8	891.48698	446.24713	297.83384	919.48189	460.24458	307.16548	F	393.24965	197.12846	131.75473	3
9	990.55540	495.78134	330.85665	1018.55031	509.77879	340.18829	V	246.18123	123.59425	82.73193	2
10							K	147.11281	74.06004	49.70912	1

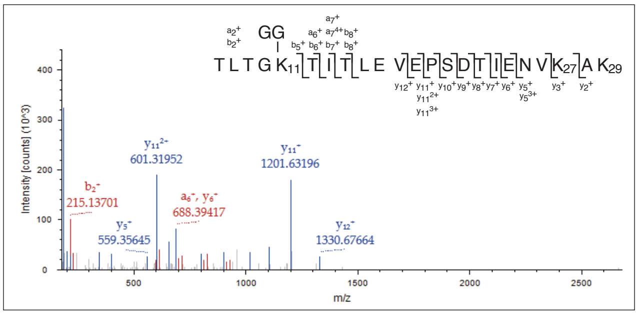

Peptide Summary

Sequence: TLTGKTITLEVEPSDTIENVKAK, K5-GG (114.04293 Da)
Charge: +4, Monoisotopic m/z: 651.10602 Da (+0.99 mmu/+1.53 ppm), MH+: 2601.40224 Da, RT: 54.59 min,
Identified with: Mascot (v1.30); IonScore:57, Exp Value:3.7E-005, Ions matched by search engine: 8/248
Fragment match tolerance used for search: 20 mmu

Fragment Matches

Value Type: Theo. Mass [Da]

Ion Series | Neutral Losses | Precursor Ions

#1	a+	a2+	a3+	a4+	b+	b2+	b3+	b4+	Seq.	y+	y2+	y3+	y4+	#2
1	74.06004	37.53366	25.35820	19.27047	102.05496	51.53112	34.68584	26.26520	T					23
2	187.14411	94.07569	63.05289	47.54149	215.13903	108.07315	72.38453	54.54021	L	2500.35059	1250.67893	834.12171	625.84310	22
3	288.19179	144.59953	96.73545	72.80341	316.18671	158.59699	106.06709	79.80213	T	2387.26652	1194.13690	796.42702	597.57209	21
4	345.21326	173.11027	115.74261	87.05877	373.20818	187.10773	125.07424	94.05750	G	2286.21884	1143.61306	762.74446	572.31017	20
5	587.35116	294.17922	196.45524	147.59325	615.34607	308.17668	205.78688	154.59198	K-GG	2229.19737	1115.10232	743.73731	558.05480	19
6	688.39894	344.70306	230.13780	172.85517	716.39375	358.70052	239.46944	179.85390	T	1987.05947	994.03337	663.02467	497.52033	18
7	801.48291	401.24509	267.83249	201.12618	829.47782	415.24255	277.16413	208.12491	I	1886.01179	943.50953	629.34211	472.25841	17
8	902.53059	451.76893	301.51505	226.38810	930.52550	465.76639	310.84669	233.38683	T	1772.92772	886.96750	591.64742	443.98739	16
9	1015.61466	508.31097	339.20974	254.65912	1043.60957	522.30843	348.54138	261.65785	L	1671.88004	836.44366	557.96486	418.72547	15
10	1144.65726	572.83227	382.22394	286.91977	1172.65217	586.82973	391.55558	293.91850	E	1558.79597	779.90162	520.27017	390.45445	14
11	1243.72568	622.36648	415.24674	311.68688	1271.72059	636.36394	424.57838	318.68561	V	1429.75337	715.38032	477.25597	358.19080	13
12	1372.76828	686.88778	458.26094	343.94753	1400.76319	700.88524	467.59258	350.94626	E	1330.68495	665.84611	444.23317	333.42670	12
13	1469.82105	735.41416	490.61187	368.21072	1497.81596	749.41162	499.94351	375.20945	P	1201.64236	601.32481	401.21897	301.16605	11
14	1556.85308	778.93018	519.62254	389.96873	1584.84799	792.92764	528.95418	396.96746	S	1104.58958	552.79643	368.86804	276.90295	10
15	1671.88003	836.44365	557.96486	418.72546	1699.87494	850.44111	567.29650	425.72419	D	1017.55756	509.28241	339.85737	255.14485	9
16	1772.92771	886.96749	591.64742	443.98738	1800.92262	900.96495	600.97906	450.98611	T	902.53060	451.76894	301.51506	226.38811	8
17	1886.01178	943.50953	629.34211	472.25840	1914.00669	957.50699	638.67375	479.25713	I	801.48292	401.24510	267.83249	201.12619	7
18	2015.05438	1008.03083	672.35631	504.51905	2043.04929	1022.02829	681.68795	511.51778	E	688.39885	344.70306	230.13780	172.85517	6
19	2129.09731	1065.05229	710.37062	533.02978	2157.09222	1079.04975	719.70226	540.02851	N	559.35625	280.18176	187.12360	140.59452	5
20	2228.16573	1114.58650	743.39343	557.79689	2256.16064	1128.58396	752.72507	564.79562	V	445.31332	223.16030	149.10929	112.08379	4
21	2356.26070	1178.63399	786.09175	589.82063	2384.25561	1192.83145	795.42339	596.81936	K	346.24490	173.62609	116.08648	87.31668	3
22	2427.29782	1214.15255	809.77079	607.57991	2455.29273	1228.15001	819.10243	614.57864	A	218.14993	109.57860	73.38816	55.29294	2
23									K	147.11281	74.06004	49.70912	37.53366	1

C2

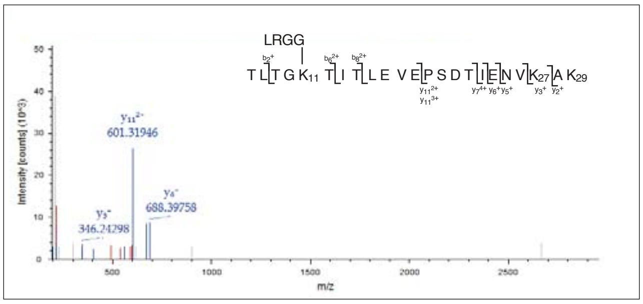

Peptide Summary
Sequence: TLTGKTITLEVEPSDTIENVKAK, K5-LRGG (383.22810 Da)
Charge: +5, Monoisotopic m/z: 574.91291 Da (+0.4 mmu/+0.7 ppm), MH+: 2870.56546 Da, RT: 52.52 min.
Identified with: Mascot (v1.30); IonScore:13, Exp Value:7.0E-001, Ions matched by search engine: 6/248
Fragment match tolerance used for search: 20 mmu

Fragment Matches

[illegible]

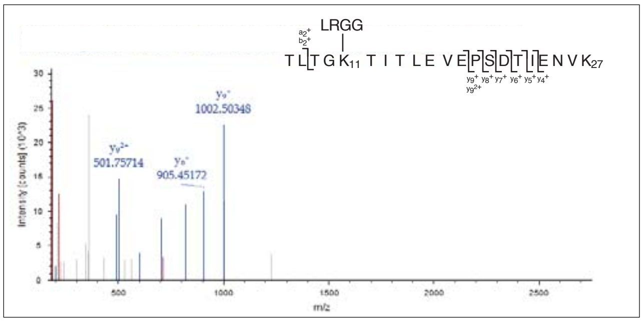

Peptide Summary
Sequence: TLTGKTITLEVEPSDTIENVK, K5-LRGG (383.22810 Da)
Charge: +4, Monoisotopic m/z: 668.61798 Da (-0.32 mmu/-0.47 ppm), MH+: 2671.45009 Da, RT: 55.73 min.
Identified with: Mascot (v1.30); IonScore:28, Exp Value:2.8E-002, Ions matched by search engine: 5/224
Fragment match tolerance used for search: 20 mmu

Fragment Matches

[illegible]

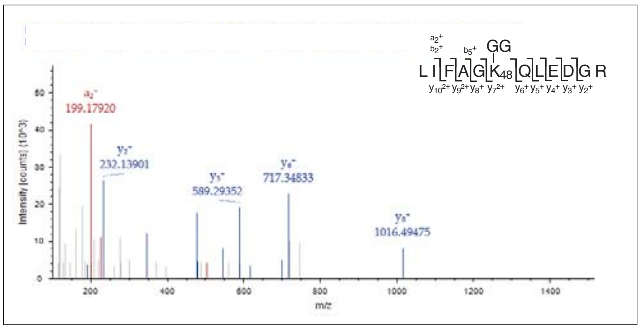

Peptide Summary
Sequence: LIFAGKQLEDGR, K6-GG (114.04293 Da)
Charge: +3, Monoisotopic m/z: 487.60025 Da (+0.17 mmu/+0.36 ppm), MH+: 1460.78620 Da, RT: 46.52 min,
Identified with: Mascot (v1.30); IonScore:58, Exp Value:4.6E-005, Ions matched by search engine: 6/112
Fragment match tolerance used for search: 20 mmu

Fragment Matches
Value Type: Theo. Mass [Da]
Ion Series | Neutral Losses | Precursor Ions

#1	a+	a2+	a3+	b+	b2+	b3+	Seq.	y+	y2+	y3+	#2
1	86.09643	43.55185	29.37033	114.09135	57.54931	38.70197	L				12
2	199.18050	100.09389	67.06502	227.17542	114.09135	76.39666	I	1347.70161	674.35444	449.90539	11
3	346.24892	173.62810	116.08783	374.24384	187.62556	125.41946	F	1234.61754	617.81241	412.21070	10
4	417.28604	209.14666	139.76687	445.28096	223.14412	149.09850	A	1087.54912	544.27820	363.18789	9
5	474.30751	237.65739	158.77402	502.30243	251.65485	168.10566	G	1016.51200	508.75964	339.50885	8
6	716.44541	358.72634	239.48665	744.44032	372.72380	248.81829	K-GG	959.49053	480.24890	320.50169	7
7	844.50399	422.75563	282.17285	872.49890	436.75309	291.50449	Q	717.35263	359.17995	239.78906	6
8	957.58806	479.29767	319.86754	985.58297	493.29513	329.19918	L	589.29405	295.15066	197.10287	5
9	1086.63066	543.81897	362.88174	1114.62557	557.81643	372.21338	E	476.20998	238.60863	159.40818	4
10	1201.65761	601.33244	401.22405	1229.65252	615.32990	410.55569	D	347.16738	174.08733	116.39398	3
11	1258.67908	629.84318	420.23121	1286.67399	643.84064	429.56285	G	232.14043	116.57385	78.05166	2
12							R	175.11896	88.06312	59.04450	1

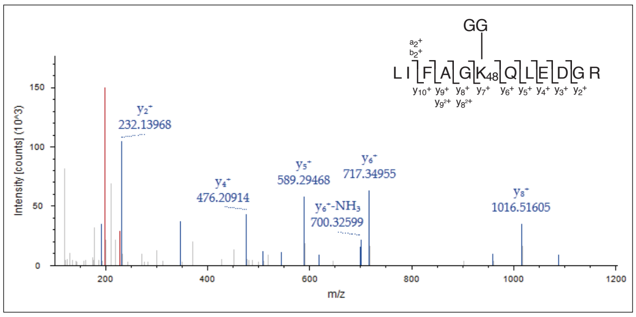

Peptide Summary
Sequence: LIFAGKQLEDGR, K6-GG (114.04293 Da)
Charge: +3, Monoisotopic m/z: 487.60056 Da (+0.48 mmu/+0.98 ppm), MH+: 1460.78711 Da, RT: 46.75 min,
Identified with: Mascot (v1.30); IonScore:60, Exp Value:2.4E-005, Ions matched by search engine: 7/112
Fragment match tolerance used for search: 20 mmu

Fragment Matches
Value Type: Theo. Mass [Da]
Ion Series | Neutral Losses | Precursor Ions

#1	a+	a2+	a3+	b+	b2+	b3+	Seq.	y+	y2+	y3+	#2
1	86.09643	43.55185	29.37033	114.09135	57.54931	38.70197	L				12
2	199.18050	100.09389	67.06502	227.17542	114.09135	76.39666	I	1347.70161	674.35444	449.90539	11
3	346.24892	173.62810	116.08783	374.24384	187.62556	125.41946	F	1234.61754	617.81241	412.21070	10
4	417.28604	209.14666	139.76687	445.28096	223.14412	149.09850	A	1087.54912	544.27820	363.18789	9
5	474.30751	237.65739	158.77402	502.30243	251.65485	168.10566	G	1016.51200	508.75964	339.50885	8
6	716.44541	358.72634	239.48665	744.44032	372.72380	248.81829	K-GG	959.49053	480.24890	320.50169	7
7	844.50399	422.75563	282.17285	872.49890	436.75309	291.50449	Q	717.35263	359.17995	239.78906	6
8	957.58806	479.29767	319.86754	985.58297	493.29513	329.19918	L	589.29405	295.15066	197.10287	5
9	1086.63066	543.81897	362.88174	1114.62557	557.81643	372.21338	E	476.20998	238.60863	159.40818	4
10	1201.65761	601.33244	401.22405	1229.65252	615.32990	410.55569	D	347.16738	174.08733	116.39398	3
11	1258.67908	629.84318	420.23121	1286.67399	643.84064	429.56285	G	232.14043	116.57385	78.05166	2
12							R	175.11896	88.06312	59.04450	1

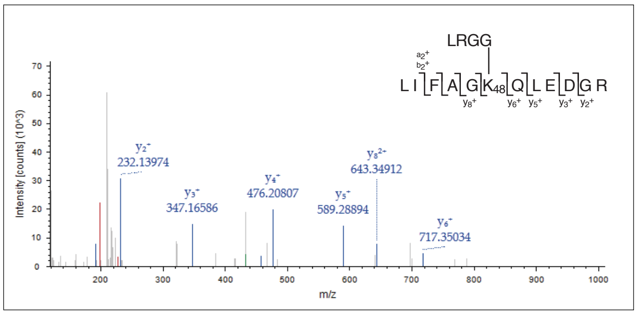

Peptide Summary
Sequence: LIFAGKQLEDGR, K6-LRGG (383.22810 Da)
Charge: +4, Monoisotopic m/z: 433.24872 Da (+0.55 mmu/+1.27 ppm), MH+: 1729.97304 Da, RT: 45.51 min,
Identified with: Mascot (v1.30); IonScore:19, Exp Value:3.1E-001, Ions matched by search engine: 4/112
Fragment match tolerance used for search: 20 mmu

Fragment Matches
Value Type: Theo. Mass [Da]
Ion Series | Neutral Losses | Precursor Ions

#1	a+	a2+	a3+	a4+	b+	b2+	b3+	b4+	Seq.	y+	y2+	y3+	y4+	#2
1	86.09643	43.55185	29.37033	22.27957	114.09135	57.54931	38.70197	29.27829	L					12
2	199.18050	100.09389	67.06502	50.55058	227.17542	114.09135	76.39666	57.54931	I	1616.88678	808.94703	539.63378	404.97715	11
3	346.24892	173.62810	116.08783	87.31769	374.24384	187.62556	125.41946	94.31642	F	1503.80271	752.40500	501.93909	376.70614	10
4	417.28604	209.14666	139.76687	105.07697	445.28096	223.14412	149.09850	112.07570	A	1356.73429	678.87079	452.91628	339.93903	9
5	474.30751	237.65739	158.77402	119.33234	502.30243	251.65485	168.10566	126.33106	G	1285.69717	643.35223	429.23724	322.17975	8
6	985.63059	493.31893	329.21505	247.16310	1013.62550	507.31639	338.54668	254.16183	K-LRGG	1228.67570	614.84149	410.23009	307.92438	7
7	1113.68917	557.34822	371.90124	279.17775	1141.68408	571.34568	381.23288	286.17648	Q	717.35263	359.17995	239.78906	180.09362	6
8	1226.77324	613.89026	409.59593	307.44877	1254.76815	627.88771	418.92757	314.44749	L	589.29405	295.15066	197.10287	148.07897	5
9	1355.81584	678.41156	452.61013	339.70942	1383.81075	692.40901	461.94177	346.70814	E	476.20998	238.60863	159.40818	119.80795	4
10	1470.84279	735.92503	490.95245	368.46615	1498.83770	749.92249	500.28408	375.46488	D	347.16738	174.08733	116.39398	87.54730	3
11	1527.86426	764.43577	509.95960	382.72152	1555.85917	778.43322	519.29124	389.72025	G	232.14043	116.57385	78.05166	58.79057	2
12									R	175.11896	88.06312	59.04450	44.53520	1

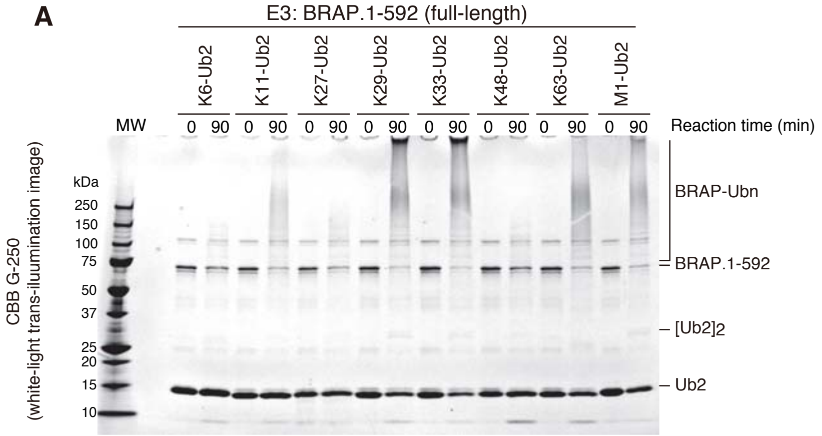
A
E3: BRAP.1-592 (full-length)
K6-Ub2
K11-Ub2
K27-Ub2
K29-Ub2
K33-Ub2
K48-Ub2
K63-Ub2
M1-Ub2
MW
0 90 0 90 0 90 0 90 0 90 0 90 0 90 0 90
Reaction time (min)
CBB G-250
(white-light trans-illumination image)
kDa
250
150
100
75
50
37
25
20
15
10
BRAP-Ubn
BRAP.1-592
[Ub2]2
Ub2

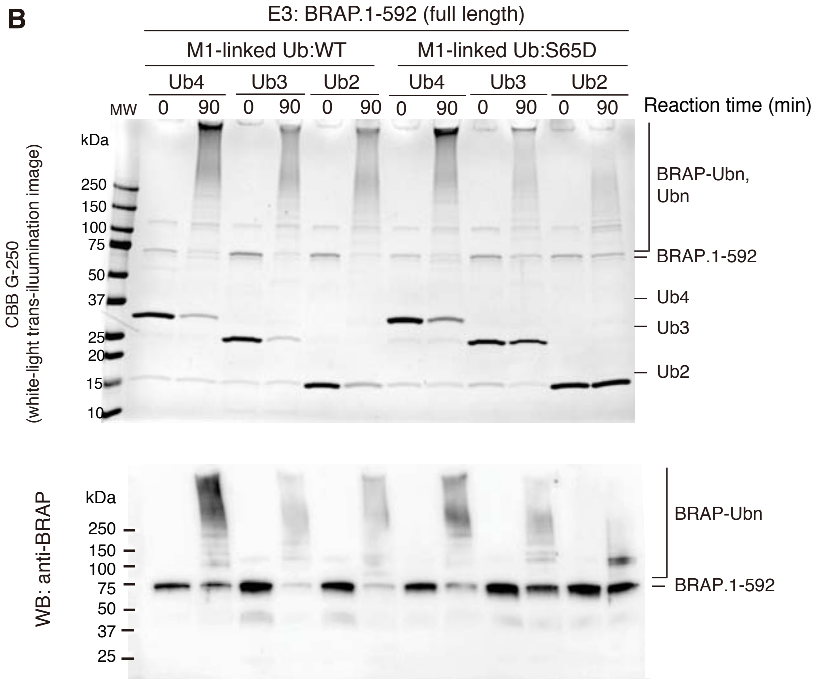
B
E3: BRAP.1-592 (full length)
M1-linked Ub:WT
M1-linked Ub:S65D
Ub4
Ub3
Ub2
Ub4
Ub3
Ub2
MW
0 90 0 90 0 90 0 90 0 90 0 90
Reaction time (min)
CBB G-250
(white-light trans-illumination image)
kDa
250
150
100
75
50
37
25
20
15
10
BRAP-Ubn,
Ubn
BRAP.1-592
Ub4
Ub3
Ub2
WB: anti-BRAP
kDa
250
150
100
75
50
37
25
BRAP-Ubn
BRAP.1-592

BCJ20161104_**Supplementary Data S7**

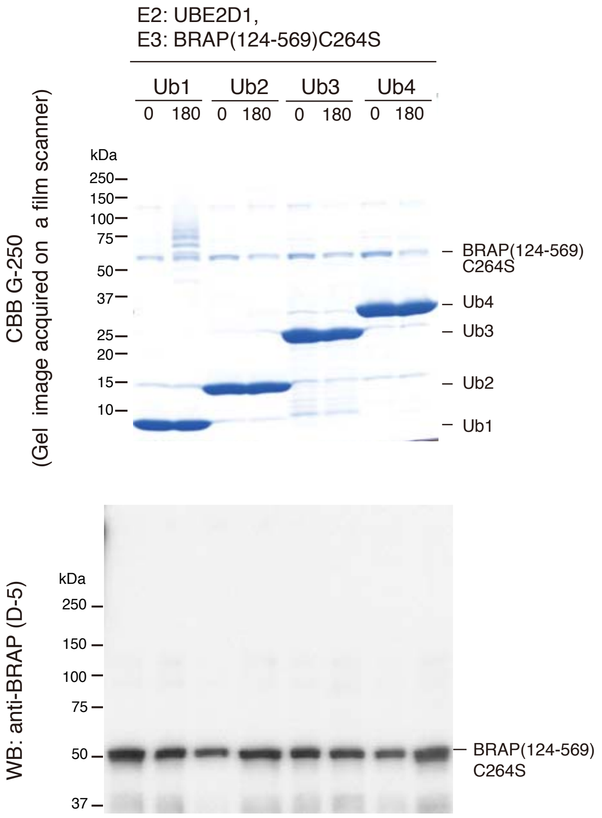

A

[M1-Ub2]$_n$, n>2

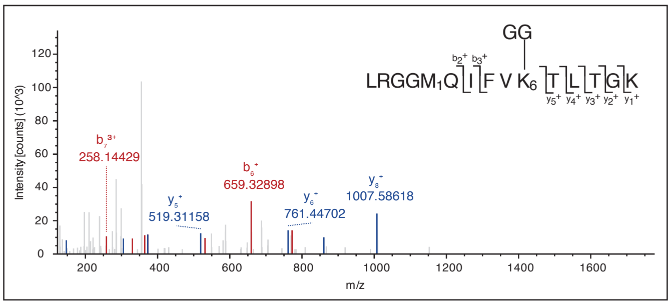
GG
LRGGM1Q I F V K6 T L T G K
b2+ b3+
y5+ y4+ y3+ y2+ y1+
b7 3+
258.14429
b6+
659.32898
y5+
519.31158
y6+
761.44702
y8+
1007.58618
Intensity [counts] (10^3)
m/z

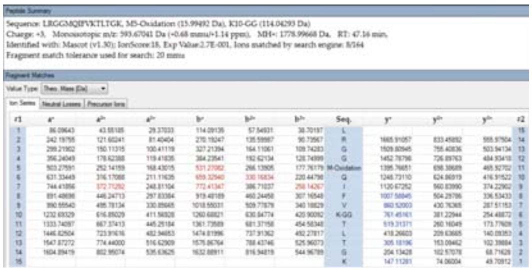
Peptide Summary
Sequence: LRGGMQIFVKTLTGK, M5-Oxidation (15.99492 Da), K10-GG (114.04293 Da)
Charge: +3, Monoisotopic m/z: 593.67041 Da (+0.68 mmu/+1.14 ppm), MH+: 1778.99668 Da, RT: 47.16 min,
Identified with: Mascot (v1.30); IonScore:18, Exp Value:2.7E-001, Ions matched by search engine: 8/164
Fragment match tolerance used for search: 20 mmu
Fragment Matches

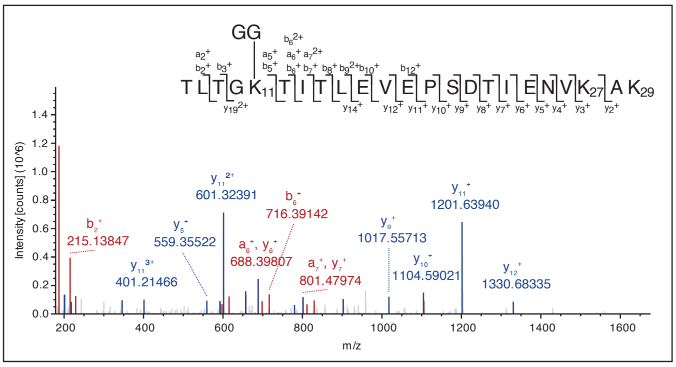
GG
T L T G K11 T I T L E V E P S D T I E N V K27 A K29
b2+
215.13847
y5+
559.35522
y11 3+
401.21466
y11 2+
601.32391
b6+
716.39142
a6+, y6+
688.39807
a7+, y7+
801.47974
y9+
1017.55713
y11+
1201.63940
y10+
1104.59021
y12+
1330.68335
Intensity [counts] (10^6)
m/z

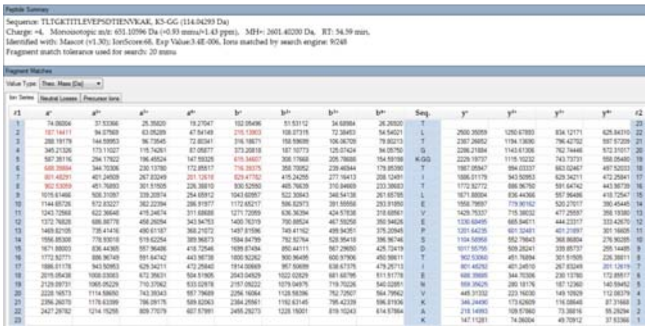
Peptide Summary
Sequence: TLTGKTITLEVEPSDTIENVKAK, K5-GG (114.04293 Da)
Charge: +4, Monoisotopic m/z: 651.10596 Da (+0.93 mmu/+1.43 ppm), MH+: 2601.40200 Da, RT: 54.59 min,
Identified with: Mascot (v1.30); IonScore:68, Exp Value:3.4E-006, Ions matched by search engine: 9/248
Fragment match tolerance used for search: 20 mmu
Fragment Matches

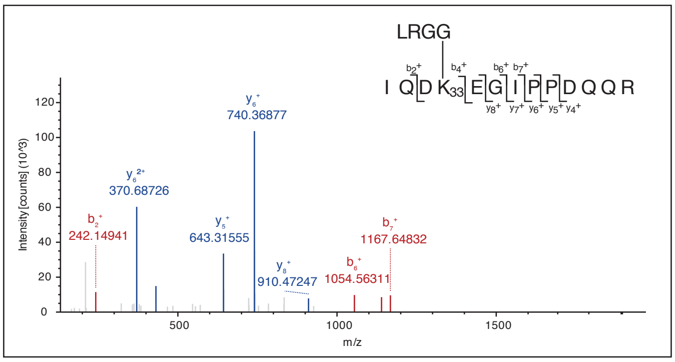
LRGG
I Q D K33 E G I P P D Q Q R
y6+
740.36877
y6 2+
370.68726
b2+
242.14941
y5+
643.31555
y8+
910.47247
b7+
1167.64832
b6+
1054.56311
Intensity [counts] (10^3)
m/z

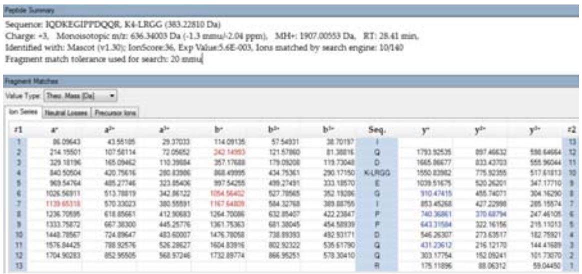
Peptide Summary
Sequence: IQDKEGIPPDQQR, K4-LRGG (383.22810 Da)
Charge: +3, Monoisotopic m/z: 636.34003 Da (-1.3 mmu/-2.04 ppm), MH+: 1907.00553 Da, RT: 28.41 min,
Identified with: Mascot (v1.30); IonScore:36, Exp Value:5.6E-003, Ions matched by search engine: 10/140
Fragment match tolerance used for search: 20 mmu
Fragment Matches

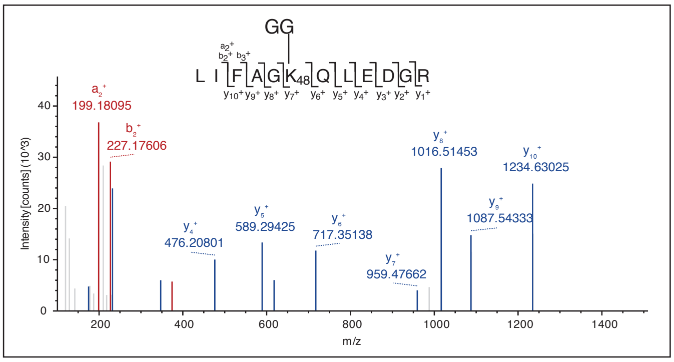
GG
L I F A G K48 Q L E D G R
a2+
199.18095
b2+
227.17606
y4+
476.20801
y5+
589.29425
y6+
717.35138
y7+
959.47662
y8+
1016.51453
y9+
1087.54333
y10+
1234.63025
Intensity [counts] (10^3)
m/z

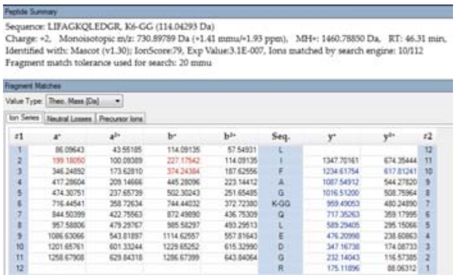
Peptide Summary
Sequence: LIFAGKQLEDGR, K6-GG (114.04293 Da)
Charge: +2, Monoisotopic m/z: 730.89789 Da (+1.41 mmu/+1.93 ppm), MH+: 1460.78850 Da, RT: 46.31 min,
Identified with: Mascot (v1.30); IonScore:79, Exp Value:3.1E-007, Ions matched by search engine: 10/112
Fragment match tolerance used for search: 20 mmu
Fragment Matches

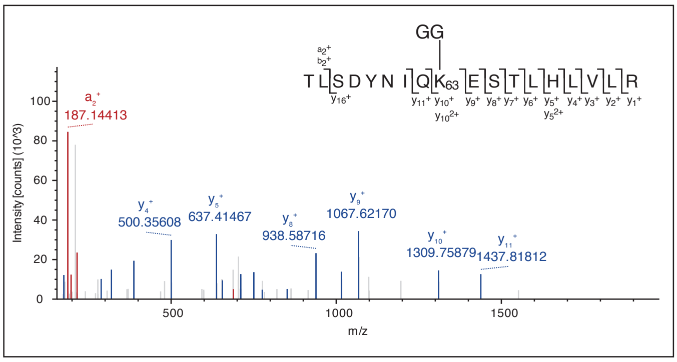
GG
T L S D Y N I Q K63 E S T L H L V L R
a2+
187.14413
y4+
500.35608
y5+
637.41467
y8+
938.58716
y9+
1067.62170
y10+
1309.75879
y11+
1437.81812
Intensity [counts] (10^3)
m/z

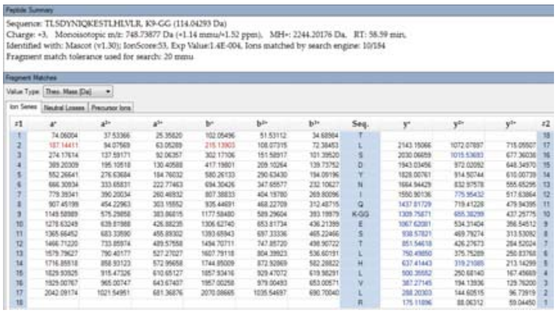
Peptide Summary
Sequence: TLSDYNIQKESTLHLVLR, K9-GG (114.04293 Da)
Charge: +3, Monoisotopic m/z: 748.73877 Da (+1.14 mmu/+1.52 ppm), MH+: 2244.20176 Da, RT: 58.59 min,
Identified with: Mascot (v1.30); IonScore:53, Exp Value:1.4E-004, Ions matched by search engine: 10/184
Fragment match tolerance used for search: 20 mmu
Fragment Matches

B

[M1-Ub3]n

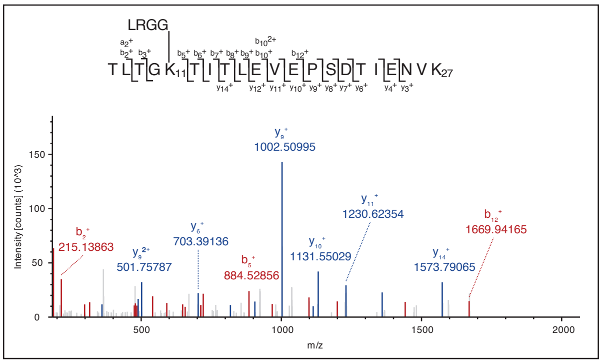

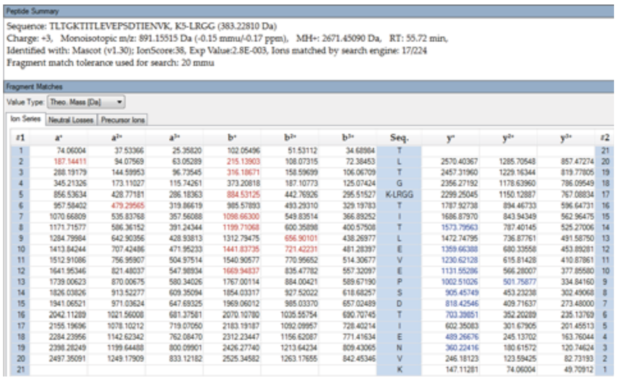

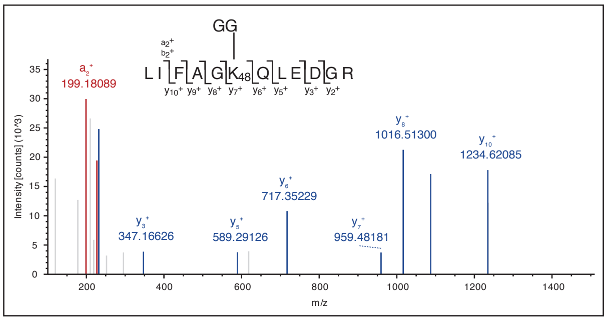

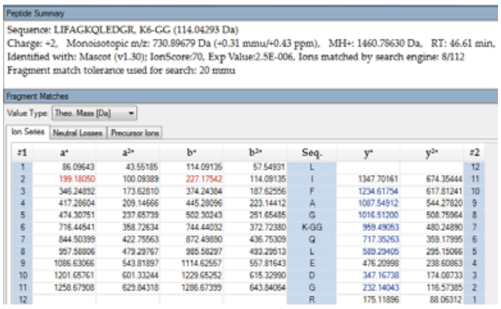

C

[M1-Ub4]n

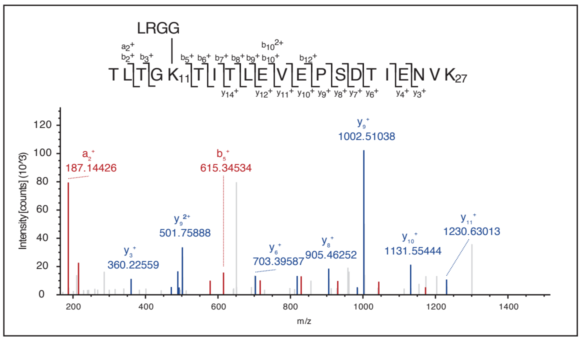

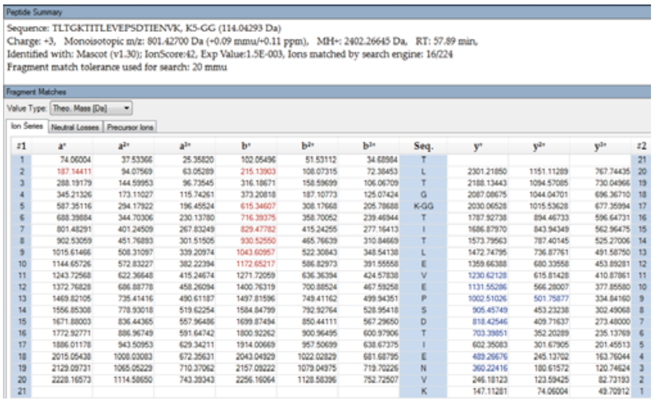

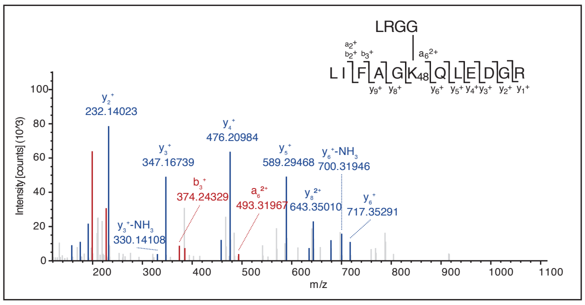

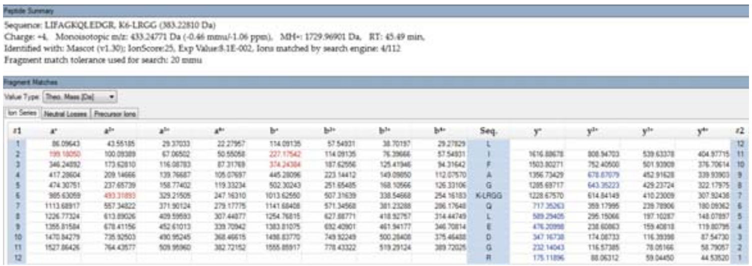

D

[M1-Ub2]2

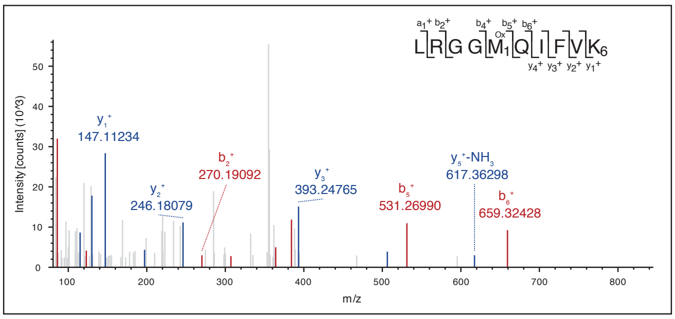

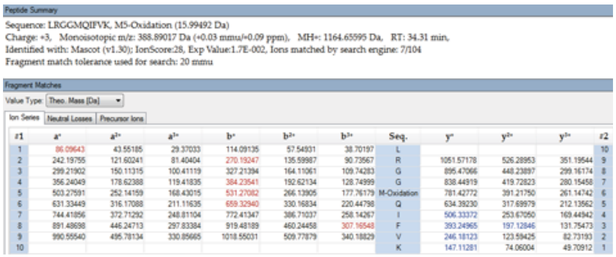

#1	a+	a2+	a3+	b+	b2+	b3+	Seq.	y+	y2+	y3+	#2
1	86.09643	43.55185	29.37033	114.09135	57.54931	38.70197	L				10
2	242.19755	121.60241	81.40404	270.19247	135.59987	90.73567	R	1051.57178	526.28953	351.19544	9
3	299.21902	150.11315	100.41119	327.21394	164.11061	109.74283	G	895.47066	448.23897	299.16174	8
4	356.24049	178.62388	119.41835	384.23541	192.62134	128.74999	G	838.44919	419.72823	280.15458	7
5	503.27591	252.14159	168.43015	531.27082	266.13905	177.76179	M-Oxidation	781.42772	391.21750	261.14742	6
6	631.33449	316.17088	211.11635	659.32940	330.16834	220.44798	Q	634.39230	317.69979	212.13562	5
7	744.41856	372.71292	248.81104	772.41347	386.71037	258.14267	I	506.33372	253.67050	169.44942	4
8	891.48698	446.24713	297.83384	919.48189	460.24458	307.16548	F	393.24965	197.12846	131.75473	3
9	990.55540	495.78134	330.85665	1018.55031	509.77879	340.18829	V	246.18123	123.59425	82.73193	2
10							K	147.11281	74.06004	49.70912	1

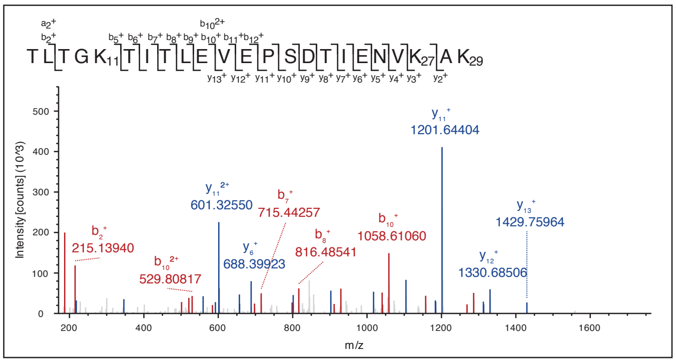

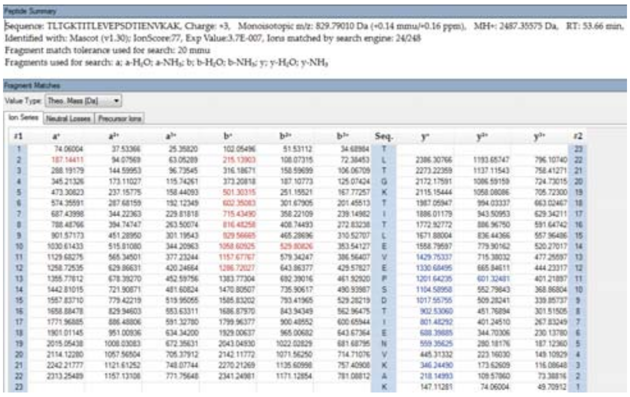

#1	a+	a2+	a3+	b+	b2+	b3+	Seq.	y+	y2+	y3+	#2
1	74.06004	37.53366	25.35820	102.05496	51.53112	34.68984	T				23
2	187.14411	94.07569	63.05289	215.13903	108.07315	72.38453	L	2386.30766	1193.65747	796.10740	22
3	288.19179	144.59953	96.73545	316.18671	158.59699	106.06709	T	2273.22359	1137.11543	758.41271	21
4	345.21326	173.11027	115.74261	373.20818	187.10773	125.07424	G	2172.17591	1086.59159	724.73015	20
5	473.30823	237.15775	158.44093	501.30315	251.15521	167.77257	K	2115.15444	1058.08086	705.72300	19
6	574.35591	287.68159	192.12349	602.35083	301.67905	201.45513	T	1987.05947	994.03337	663.02467	18
7	687.43998	344.22363	229.81818	715.43490	358.22109	239.14982	I	1886.01179	943.50953	629.34211	17
8	788.48766	394.74747	263.50074	816.48258	408.74493	272.83238	T	1772.92772	886.96750	591.64742	16
9	901.57173	451.28950	301.19543	929.56665	465.28696	310.52707	L	1671.88004	836.44366	557.96486	15
10	1030.61433	515.81080	344.20963	1058.60925	529.80826	353.54127	E	1558.79597	779.90162	520.27017	14
11	1129.68275	565.34501	377.23244	1157.67767	579.34247	386.56407	V	1429.75337	715.38032	477.25597	13
12	1258.72535	629.86631	420.24664	1286.72027	643.86377	429.57827	E	1330.68495	665.84611	444.23317	12
13	1355.77812	678.39270	452.59756	1383.77304	692.39016	461.92920	P	1201.64235	601.32481	401.21897	11
14	1442.81015	721.90871	481.60824	1470.80507	735.90617	490.93987	S	1104.58958	552.79843	368.86804	10
15	1557.83710	779.42219	519.95055	1585.83202	793.41965	529.28219	D	1017.55756	509.28241	339.85737	9
16	1658.88478	829.94603	553.63311	1686.87970	843.94349	562.96475	T	902.53060	451.76894	301.51505	8
17	1771.96885	886.48806	591.32780	1799.96377	900.48552	600.65944	I	801.48292	401.24510	267.83249	7
18	1901.01145	951.00936	634.34200	1929.00637	965.00682	643.67364	E	688.39885	344.70306	230.13780	6
19	2015.05438	1008.03083	672.35631	2043.04930	1022.02829	681.68795	N	559.35625	280.18176	187.12360	5
20	2114.12280	1057.56504	705.37912	2142.11772	1071.56250	714.71076	V	445.31332	223.16030	149.10929	4
21	2242.21777	1121.61252	748.07744	2270.21269	1135.60998	757.40908	K	346.24490	173.62609	116.08648	3
22	2313.25489	1157.13108	771.75648	2341.24981	1171.12854	781.08812	A	218.14993	109.57860	73.38816	2
23							K	147.11281	74.06004	49.70912	1

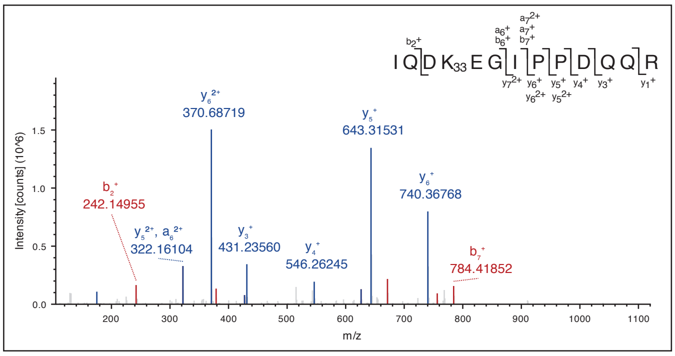

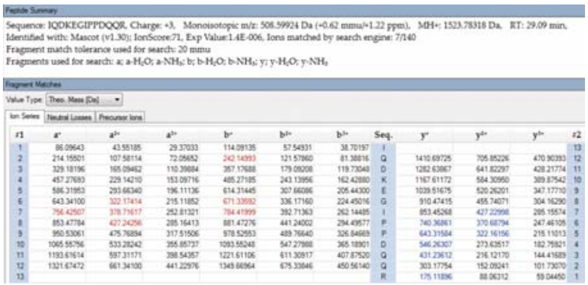

#1	a+	a2+	a3+	b+	b2+	b3+	Seq.	y+	y2+	y3+	#2
1	86.09643	43.55185	29.37033	114.09135	57.54931	38.70197	I				13
2	214.15501	107.58114	72.05652	242.14993	121.57860	81.38816	Q	1410.69725	705.85226	470.90393	12
3	329.18196	165.09462	110.39884	357.17688	179.09208	119.73048	D	1282.63867	641.82297	428.21774	11
4	457.27693	229.14210	153.09716	485.27185	243.13956	162.42880	K	1167.61172	584.30950	389.87542	10
5	586.31953	293.66340	196.11136	614.31445	307.66086	205.44300	E	1039.51675	520.26201	347.17710	9
6	643.34100	322.17414	215.11852	671.33592	336.17160	224.45016	G	910.47415	455.74071	304.16290	8
7	756.42507	378.71617	252.81321	784.41999	392.71363	262.14485	I	853.45268	427.22998	285.15574	7
8	853.47784	427.24256	285.16413	881.47276	441.24002	294.49577	P	740.36861	370.68794	247.46105	6
9	950.53061	475.76894	317.51506	978.52553	489.76640	326.84669	P	643.31584	322.16156	215.11013	5
10	1065.55756	533.28242	355.85737	1093.55248	547.27988	365.18901	D	546.26307	273.63517	182.75921	4
11	1193.61614	597.31171	398.54357	1221.61106	611.30917	407.87520	Q	431.23612	216.12170	144.41689	3
12	1321.67472	661.34100	441.22976	1349.66964	675.33846	450.56140	Q	303.17754	152.09241	101.73070	2
13							R	175.11896	88.06312	59.04450	1

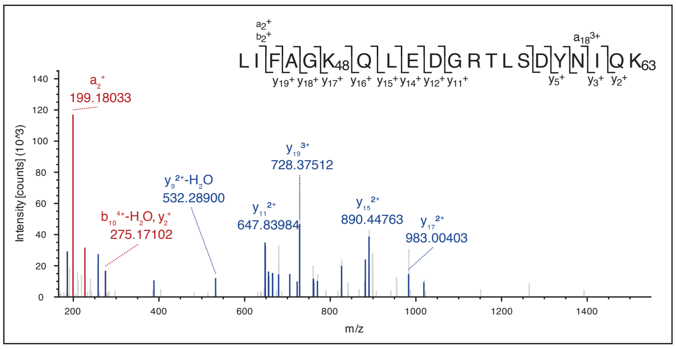

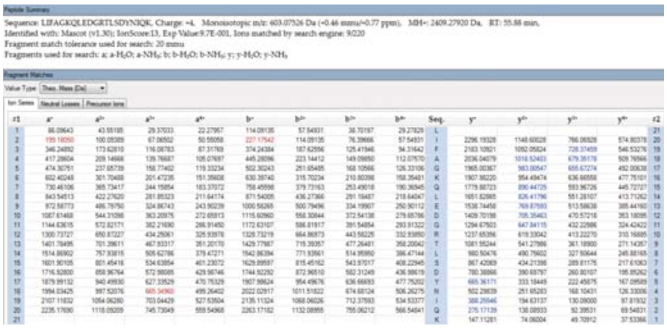

#1	a+	a2+	a3+	a4+	b+	b2+	b3+	b4+	Seq.	y+	y2+	y3+	y4+	#2
1	86.09643	43.55185	29.37033	22.27957	114.09135	57.54931	38.70197	29.27829	L					21
2	199.18050	100.09389	67.06502	50.55058	227.17542	114.09135	76.39666	57.54931	I	2296.19328	1148.60028	766.06928	574.80378	20
3	346.24892	173.62810	116.08783	87.31769	374.24384	187.62556	125.41946	94.31642	F	2183.10921	1092.05824	728.37459	546.53276	19
4	417.28604	209.14666	139.76687	105.07697	445.28096	223.14412	149.09850	112.07570	A	2036.04079	1018.52403	679.35178	509.76566	18
5	474.30751	237.65739	158.77402	119.33234	502.30243	251.65485	168.10566	126.33106	G	1965.00367	983.00547	655.67274	492.00638	17
6	602.40248	301.70488	201.47235	151.35608	630.39740	315.70234	210.80398	158.35481	K	1907.98220	954.49474	636.66558	477.75101	16
7	730.46106	365.73417	244.15854	183.37072	758.45598	379.73163	253.49018	190.36945	Q	1779.88723	890.44725	593.96726	445.72727	15
8	843.54513	422.27620	281.85323	211.64174	871.54005	436.27366	291.18487	218.64047	L	1651.82985	826.41796	551.28107	413.71262	14
9	972.58773	486.79750	324.86743	243.90239	1000.58265	500.79496	334.19907	250.90112	E	1538.74458	769.87593	513.58638	385.44160	13
10	1087.61468	544.31098	363.20975	272.65913	1115.60960	558.30844	372.54138	279.65786	D	1409.70198	705.35463	470.57218	353.18095	12
11	1144.63615	572.82171	382.21690	286.91450	1172.63107	586.81917	391.54854	293.91322	G	1294.67503	647.84115	432.22986	324.42422	11
12	1300.73727	650.87227	434.25061	325.93978	1328.73219	664.86973	443.58225	332.93850	R	1237.65356	619.33042	413.22270	310.16885	10
13	1401.78495	701.39611	467.93317	351.20170	1429.77987	715.39357	477.26481	358.20042	T	1081.55244	541.27986	361.18900	271.14357	9
14	1514.86902	757.93815	505.62786	379.47271	1542.86394	771.93561	514.95950	386.47144	L	980.50476	490.75602	327.50644	245.88165	8
15	1601.90105	801.45416	534.63854	401.23072	1629.89597	815.45162	543.97017	408.22945	S	867.42069	434.21398	289.81175	217.61063	7
16	1716.92800	858.96764	572.98085	429.98746	1744.92292	872.96510	582.31249	436.98619	D	780.38866	390.69797	260.80107	195.85262	6
17	1879.99132	940.49930	627.33529	470.75329	1907.98624	954.49676	636.66693	477.75202	Y	665.36171	333.18449	222.45875	167.09589	5
18	1994.03425	997.52076	665.34960	499.26402	2022.02917	1011.51822	674.68124	506.26275	N	502.29839	251.65283	168.10431	126.33006	4
19	2107.11832	1054.06280	703.04429	527.53504	2135.11324	1068.06026	712.37593	534.53377	I	388.25546	194.63137	130.09000	97.81932	3
20	2235.17690	1118.09209	745.73049	559.54968	2263.17182	1132.08955	755.06212	566.54841	Q	275.17139	138.08933	92.39531	69.54831	2
21									K	147.11281	74.06004	49.70912	37.53366	1

A1

BRAP(124-569)-[M1-Ub2]n

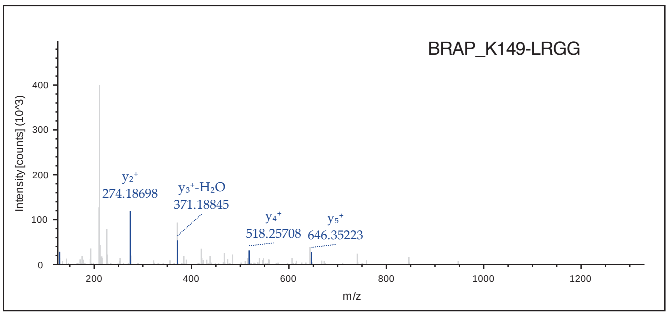

Peptide Summary

Sequence: TNKMTSLKEDVR, K3-LRGG (383.22810 Da), M4-Oxidation (15.99492 Da)
Charge: +4, Monoisotopic m/z: 455.99667 Da (+0.02 mmu/+0.05 ppm), MH+: 1820.96486 Da, RT: 24.40 min,
Identified with: Mascot (v1.30); IonScore:19, Exp Value:2.6E-001, Ions matched by search engine: 6/126
Fragment match tolerance used for search: 20 mmu

Fragment Matches

Value Type: Theo. Mass [Da]

Ion Series | Neutral Losses | Precursor Ions

#1	a+	a2+	a3+	a4+	b+	b2+	b3+	b4+	Seq.	y+	y2+	y3+	y4+
1	74.06004	37.53366	25.35820	19.27547	102.05496	51.53112	34.68984	26.26820	T				
2	188.10297	94.55512	63.37251	47.78120	216.09789	108.55258	72.70415	54.77993	N	1719.91710	860.46219	573.97722	430.73473
3	699.42605	350.21666	233.81353	175.61197	727.42096	364.21412	243.14517	182.61070	K-LRGG	1605.87417	803.44072	535.96291	402.22400
4	846.46146	423.73437	282.82534	212.37082	874.45637	437.73183	292.15698	219.36955	M-Oxidation	1094.55110	547.77919	365.52188	274.39323
5	947.50914	474.25821	316.50790	237.63274	975.50405	488.25567	325.83954	244.63147	T	947.51568	474.26148	316.51008	237.63438
6	1034.54117	517.77422	345.51857	259.39075	1062.53608	531.77168	354.85021	266.38948	S	846.46800	423.73764	282.82752	212.37246
7	1147.62524	574.31626	383.21326	287.66177	1175.62015	588.31372	392.54490	294.66050	L	759.43597	380.22162	253.81684	190.61445
8	1275.72021	638.36374	425.91159	319.68551	1303.71512	652.36120	435.24323	326.68424	K	646.35190	323.67959	216.12215	162.34343
9	1404.76281	702.88504	468.92579	351.94616	1432.75772	716.88250	478.25743	358.94489	E	518.25693	259.63210	173.42383	130.31969
10	1519.78976	760.39852	507.26810	380.70290	1547.78467	774.39598	516.59974	387.70163	D	389.21433	195.11080	130.40963	98.05904
11	1618.85818	809.93273	540.29091	405.47000	1646.85309	823.93019	549.62255	412.46873	V	274.18738	137.59733	92.06731	69.30230
12									R	175.11896	88.06312	59.04450	44.53520

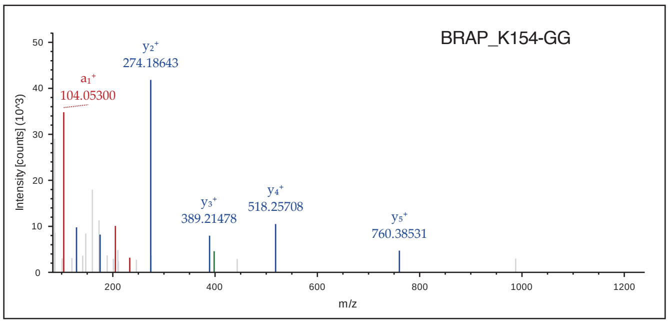

Peptide Summary

Sequence: MTSLKEDVR, K5-GG (114.04293 Da)
Charge: +3, Monoisotopic m/z: 398.20422 Da (-0.33 mmu/-0.83 ppm), MH+: 1192.59812 Da, RT: 29.69 min,
Identified with: Mascot (v1.30); IonScore:31, Exp Value:1.4E-002, Ions matched by search engine: 4/80
Fragment match tolerance used for search: 20 mmu

Fragment Matches

Value Type: Theo. Mass [Da]

Ion Series | Neutral Losses | Precursor Ions

#1	a+	a2+	a3+	b+	b2+	b3+	Seq.	y+	y2+	y3+	#2
1	104.05286	52.53007	35.35581	132.04778	66.52753	44.68744	M				9
2	205.10054	103.05391	69.03837	233.09546	117.05137	78.37000	T	1061.55861	531.28294	354.52439	8
3	292.13257	146.56992	98.04904	320.12749	160.56738	107.38068	S	960.51093	480.75910	320.84183	7
4	405.21664	203.11196	135.74373	433.21156	217.10942	145.07537	L	873.47890	437.24309	291.83115	6
5	647.35454	324.18091	216.45636	675.34945	338.17837	225.78800	K-GG	760.39483	380.70105	254.13646	5
6	776.39714	388.70221	259.47056	804.39205	402.69967	268.80220	E	518.25693	259.63210	173.42383	4
7	891.42409	446.21568	297.81288	919.41900	460.21314	307.14452	D	389.21433	195.11080	130.40963	3
8	990.49251	495.74989	330.83569	1018.48742	509.74735	340.16733	V	274.18738	137.59733	92.06731	2
9							R	175.11896	88.06312	59.04450	1

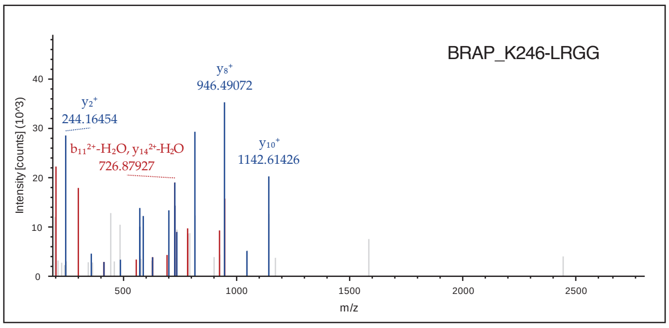

Peptide Summary

Sequence: AEVLKSEDGASLPVMDLTELPK, K5-LRGG (383.22810 Da)
Charge: +4, Monoisotopic m/z: 682.11774 Da (+1.23 mmu/+1.81 ppm), MH+: 2725.44912 Da, RT: 65.70 min,
Identified with: Mascot (v1.30); IonScore:48, Exp Value:3.2E-004, Ions matched by search engine: 9/236
Fragment match tolerance used for search: 20 mmu

Fragment Matches

Value Type: Theo. Mass [Da]

Ion Series | Neutral Losses | Precursor Ions

#1	a+	a2+	a3+	a4+	b+	b2+	b3+	b4+	Seq.	y+	y2+	y3+	y4+	#2
1	44.04948	22.52838	15.35468	11.76783	72.04440	36.52584	24.68632	18.76656	A					22
2	173.09208	87.04968	58.36888	44.02848	201.08700	101.04714	67.70052	51.02721	E	2654.40707	1327.70718	885.47388	664.35723	21
3	272.16050	136.58389	91.39169	68.79558	300.15542	150.58135	100.72332	75.79431	V	2525.36447	1263.18588	842.45968	632.09658	20
4	385.24457	193.12592	129.08638	97.06660	413.23949	207.12338	138.41801	104.06533	L	2426.29605	1213.65167	809.43687	607.32947	19
5	896.56765	448.78746	299.52740	224.89737	924.56256	462.78492	308.85904	231.89610	K-LRGG	2313.21198	1157.10963	771.74218	579.05845	18
6	983.59968	492.30348	328.53808	246.65538	1011.59459	506.30093	337.86971	253.65410	S	1801.88891	901.44809	601.30115	451.22769	17
7	1112.64228	556.82478	371.55228	278.91603	1140.63719	570.82223	380.88391	285.91475	E	1714.85688	857.93208	572.29040	429.46968	16
8	1227.66923	614.33825	409.89459	307.67276	1255.66414	628.33571	419.22623	314.67149	D	1585.81428	793.41078	529.27628	397.20903	15
9	1284.69070	642.84899	428.90175	321.92813	1312.68561	656.84644	438.23339	328.92686	G	1470.78733	735.89730	490.93396	368.45229	14
10	1355.72782	678.36755	452.58079	339.68741	1383.72273	692.36500	461.91243	346.68614	A	1413.76586	707.38657	471.92680	354.19692	13
11	1442.75985	721.88356	481.59147	361.44542	1470.75476	735.88102	490.92310	368.44415	S	1342.72874	671.86801	448.24776	336.43764	12
12	1555.84392	778.42560	519.28616	389.71644	1583.83883	792.42305	528.61779	396.71516	L	1255.69671	628.35199	419.23709	314.67964	11
13	1652.89669	826.95198	551.63708	413.97963	1680.89160	840.94944	560.96872	420.97836	P	1142.61264	571.80996	381.54240	286.40862	10
14	1751.96511	876.48619	584.65989	438.74673	1779.96002	890.48365	593.99152	445.74546	V	1045.55987	523.28357	349.19147	262.14543	9
15	1883.00561	942.00644	628.34005	471.50686	1911.00052	956.00390	637.67169	478.50559	M	946.49145	473.74936	316.16867	237.37832	8
16	1998.03256	999.51992	666.68237	500.26360	2026.02747	1013.51737	676.01401	507.26232	D	815.45095	408.22911	272.48850	204.61820	7
17	2111.11663	1056.06195	704.37706	528.53461	2139.11154	1070.05941	713.70870	535.53334	L	700.42400	350.71564	234.14618	175.86146	6
18	2212.16431	1106.58579	738.05962	553.79653	2240.15922	1120.58325	747.39126	560.79526	T	587.33993	294.17360	196.45149	147.59044	5
19	2341.20691	1171.10709	781.07382	586.05718	2369.20182	1185.10455	790.40546	593.05591	E	486.29225	243.64976	162.76893	122.32852	4
20	2454.29098	1227.64913	818.76851	614.32820	2482.28589	1241.64658	828.10015	621.32693	L	357.24965	179.12846	119.75473	90.06787	3
21	2551.34375	1276.17551	851.11943	638.59139	2579.33866	1290.17297	860.45107	645.59012	P	244.16558	122.58643	82.06004	61.79685	2
22									K	147.11281	74.06004	49.70912	37.53366	1

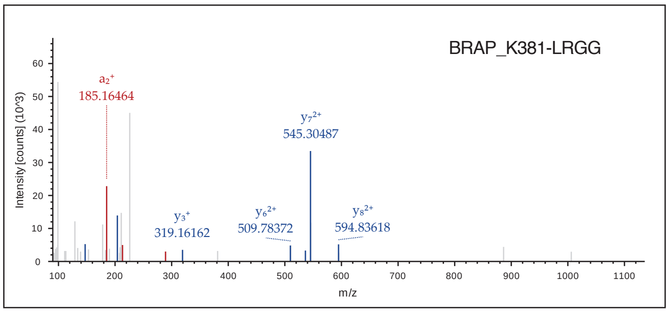

Peptide Summary

Sequence: LVASKTDGK, K5-LRGG (383.22810 Da)
Charge: +3, Monoisotopic m/z: 434.59000 Da (+0.6 mmu/+1.39 ppm), MH+: 1301.75544 Da, RT: 18.33 min,
Identified with: Mascot (v1.30); IonScore:14, Exp Value:6.1E-001, Ions matched by search engine: 6/80
Fragment match tolerance used for search: 20 mmu

Fragment Matches

Value Type: Theo. Mass [Da]

Ion Series | Neutral Losses | Precursor Ions

#1	a+	a2+	a3+	b+	b2+	b3+	Seq.	y+	y2+	y3+	#2
1	86.09643	43.55185	29.37033	114.09135	57.54931	38.70197	L				9
2	185.16485	93.08606	62.39314	213.15977	107.08352	71.72477	V	1188.66955	594.83842	396.89470	8
3	256.20197	128.60462	86.07218	284.19689	142.60208	95.40381	A	1089.60113	545.30421	363.87190	7
4	343.23400	172.12064	115.08285	371.22892	186.11810	124.41449	S	1018.56401	509.78565	340.19286	6
5	854.55708	427.78218	285.52388	882.55199	441.77963	294.85551	K-LRGG	931.53198	466.26963	311.18218	5
6	955.60476	478.30602	319.20644	983.59967	492.30347	328.53807	T	420.20891	210.60809	140.74115	4
7	1070.63171	535.81549	357.54875	1098.62662	549.81695	366.88039	D	319.16123	160.08425	107.05859	3
8	1127.65318	564.33023	376.55591	1155.64809	578.32768	385.88755	G	204.13428	102.57078	68.71628	2
9							K	147.11281	74.06004	49.70912	1

A2

BRAP(124-569)-[M1-Ub2]n

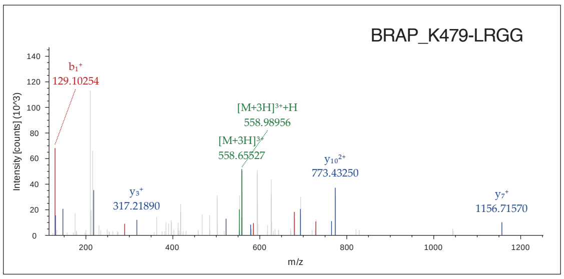

Peptide Summary

Sequence: KCTQLNTKVAK, C2-Carbamidomethyl (57.02146 Da), K8-LRGG (383.22810 Da)
Charge: +3, Monoisotopic m/z: 558.65491 Da (+0.73 mmu/+1.3 ppm), MH+: 1673.95017 Da, RT: 21.41 min,
Identified with: Mascot (v1.30); IonScore:12, Exp Value:1.3E+000, Ions matched by search engine: 9/120
Fragment match tolerance used for search: 20 mmu

Fragment Matches

Value Type: Theo. Mass [Da] | Ion Series | Neutral Losses | Precursor Ions

#1	a^+	a^{2+}	a^{3+}	b^+	b^{2+}	b^{3+}	Seq.	y^+	y^{2+}	y^{3+}	#2
1	101.10733	51.05730	34.37396	129.10225	65.05476	43.70560	K				11
2	261.13799	131.07263	87.71751	289.13290	145.07009	97.04915	C-Carbami...	1545.85302	773.43015	515.95586	10
3	362.18567	181.59647	121.40007	390.18058	195.59393	130.73171	T	1385.82236	693.41482	462.61231	9
4	490.24425	245.62576	164.08627	518.23916	259.62322	173.41790	Q	1284.77468	642.89098	428.92975	8
5	603.32832	302.16780	201.78096	631.32323	316.16525	211.11259	L	1156.71610	578.86169	386.24355	7
6	717.37125	359.18926	239.79527	745.36616	373.18672	249.12690	N	1043.63203	522.31966	348.54886	6
7	818.41893	409.71310	273.47783	846.41384	423.71056	282.80946	T	929.58910	465.29819	310.53455	5
8	1329.74200	665.37464	443.91885	1357.73691	679.37210	453.25049	K-LRGG	828.54142	414.77435	276.85199	4
9	1428.81042	714.90885	476.94166	1456.80533	728.90631	486.27330	V	317.21835	159.11281	106.41097	3
10	1499.84754	750.42741	500.62070	1527.84245	764.42487	509.95234	A	218.14993	109.57860	73.38816	2
11							K	147.11281	74.06004	49.70912	1

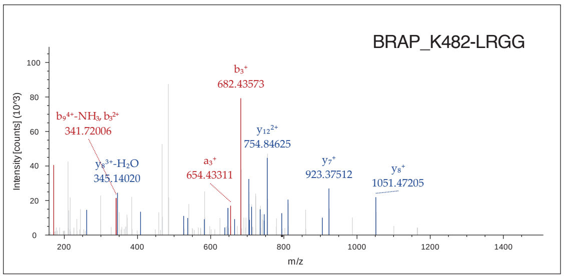

Peptide Summary

Sequence: VAKLTNELKEEQEMNK, K3-LRGG (383.22810 Da), M14-Oxidation (15.99492 Da)
Charge: +4, Monoisotopic m/z: 576.55658 Da (+0.51 mmu/+0.88 ppm), MH+: 2303.20449 Da, RT: 34.87 min,
Identified with: Mascot (v1.30); IonScore:26, Exp Value:7.3E-002, Ions matched by search engine: 13/172
Fragment match tolerance used for search: 20 mmu

Fragment Matches

Value Type: Theo. Mass [Da] | Ion Series | Neutral Losses | Precursor Ions

#1	a^+	a^{2+}	a^{3+}	a^{4+}	b^+	b^{2+}	b^{3+}	b^{4+}	Seq.	y^+	y^{2+}	y^{3+}	y^{4+}	#2
1	72.08078	36.54403	24.69845	18.77565	100.07570	50.54149	34.03008	25.77438	V					16
2	143.11790	72.06259	48.37749	36.53493	171.11282	86.06005	57.70912	43.53366	A	2204.13405	1102.57066	735.38287	551.78897	15
3	654.44098	327.72413	218.81851	164.36570	682.43589	341.72158	228.15015	171.36443	K-LRGG	2133.09693	1067.05210	711.70383	534.02969	14
4	767.52505	384.26616	256.51320	192.63672	795.51996	398.26362	265.84484	199.63545	L	1621.77386	811.39057	541.26280	406.19892	13
5	868.57273	434.79000	290.19576	217.89864	896.56764	448.78746	299.52740	224.89737	T	1508.68979	754.84853	503.56811	377.92790	12
6	982.61566	491.81147	328.21007	246.40937	1010.61057	505.80892	337.54171	253.40810	N	1407.64211	704.32469	469.88555	352.66598	11
7	1111.65826	556.33277	371.22427	278.67002	1139.65317	570.33022	380.55591	285.66875	E	1293.59918	647.30323	431.87124	324.15525	10
8	1224.74233	612.87480	408.91896	306.94104	1252.73724	626.87226	418.25060	313.93977	L	1164.55658	582.78193	388.85704	291.89460	9
9	1352.83730	676.92229	451.61728	338.96478	1380.83221	690.91974	460.94892	345.96351	K	1051.47251	526.23989	351.16235	263.62358	8
10	1481.87990	741.44359	494.63148	371.22543	1509.87481	755.44104	503.96312	378.22416	E	923.37754	462.19241	308.46403	231.59984	7
11	1610.92250	805.96489	537.64568	403.48608	1638.91741	819.96234	546.97732	410.48481	E	794.33494	397.67111	265.44983	199.33919	6
12	1738.98108	869.99418	580.33188	435.50073	1766.97599	883.99163	589.66351	442.49945	Q	665.29234	333.14981	222.43563	167.07854	5
13	1868.02368	934.51548	623.34608	467.76138	1896.01859	948.51293	632.67771	474.76010	E	537.23376	269.12052	179.74944	135.06390	4
14	2015.05909	1008.03318	672.35788	504.52023	2043.05400	1022.03064	681.68952	511.51896	M-Oxidation	408.19116	204.59922	136.73524	102.80325	3
15	2129.10202	1065.05465	710.37219	533.03096	2157.09693	1079.05211	719.70383	540.02969	N	261.15574	131.08151	87.72343	66.04439	2
16									K	147.11281	74.06004	49.70912	37.53366	1

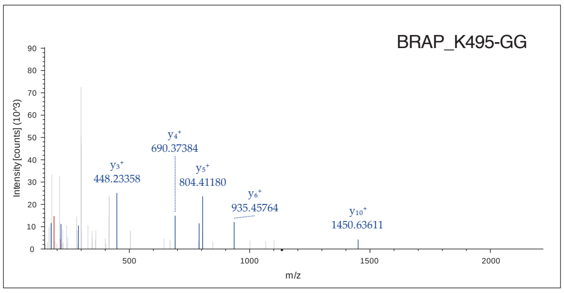

Peptide Summary

Sequence: LTNELKEEQEMNKCLR, K13-GG (114.04293 Da), C14-Carbamidomethyl (57.02146 Da)
Charge: +4, Monoisotopic m/z: 538.01544 Da (+0.56 mmu/+1.04 ppm), MH+: 2149.03994 Da, RT: 40.45 min,
Identified with: Mascot (v1.30); IonScore:18, Exp Value:3.0E-001, Ions matched by search engine: 5/172
Fragment match tolerance used for search: 20 mmu

Fragment Matches

Value Type: Theo. Mass [Da] | Ion Series | Neutral Losses | Precursor Ions

#1	a^+	a^{2+}	a^{3+}	a^{4+}	b^+	b^{2+}	b^{3+}	b^{4+}	Seq.	y^+	y^{2+}	y^{3+}	y^{4+}	#2
1	86.09643	43.55185	29.37033	22.27957	114.09135	57.54931	38.70197	29.27829	L					16
2	187.14411	94.07569	63.05289	47.54149	215.13903	108.07315	72.38453	54.54021	T	2035.95364	1018.48046	679.32273	509.74387	15
3	301.18704	151.09716	101.06720	76.05222	329.18196	165.09462	110.39884	83.05095	N	1934.90596	967.95662	645.64017	484.48195	14
4	430.22964	215.61846	144.08140	108.31287	458.22456	229.61592	153.41304	115.31160	E	1820.86303	910.93515	607.62586	455.97122	13
5	543.31371	272.16049	181.77609	136.58389	571.30863	286.15795	191.10773	143.58261	L	1691.82043	846.41385	564.61166	423.71057	12
6	671.40868	336.20798	224.47441	168.60763	699.40360	350.20544	233.80605	175.60636	K	1578.73636	789.87182	526.91697	395.43955	11
7	800.45128	400.72928	267.48861	200.86828	828.44620	414.72674	276.82025	207.86701	E	1450.64139	725.82433	484.21865	363.41581	10
8	929.49388	465.25058	310.50281	233.12893	957.48880	479.24804	319.83445	240.12766	E	1321.59879	661.30303	441.20445	331.15516	9
9	1057.55246	529.27987	353.18901	265.14357	1085.54738	543.27733	362.52064	272.14230	Q	1192.55619	596.78173	398.19025	298.89451	8
10	1186.59506	593.80117	396.20321	297.40422	1214.58998	607.79863	405.53484	304.40295	E	1064.49761	532.75244	355.50406	266.87986	7
11	1317.63556	659.32142	439.88337	330.16435	1345.63048	673.31888	449.21501	337.16308	M	935.45501	468.23114	312.48986	234.61921	6
12	1431.67849	716.34288	477.89768	358.67508	1459.67341	730.34034	487.22932	365.67381	N	804.41451	402.71089	268.80969	201.85909	5
13	1673.81639	837.41183	558.61031	419.20955	1701.81130	851.40929	567.94195	426.20828	K-GG	690.37158	345.68943	230.79538	173.34835	4
14	1833.84704	917.42716	611.95387	459.21722	1861.84196	931.42462	621.28550	466.21595	C-Carbami...	448.23369	224.62048	150.08275	112.81388	3
15	1946.93111	973.96919	649.64856	487.48824	1974.92603	987.96665	658.98019	494.48696	L	288.20303	144.60515	96.73919	72.80622	2
16									R	175.11896	88.06312	59.04450	44.53520	1

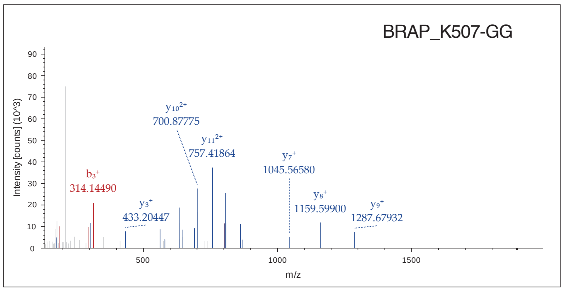

Peptide Summary

Sequence: ANQVLLQNKLKEEER, K9-GG (114.04293 Da)
Charge: +3, Monoisotopic m/z: 642.68512 Da (+0.14 mmu/+0.21 ppm), MH+: 1926.04081 Da, RT: 40.05 min,
Identified with: Mascot (v1.30); IonScore:56, Exp Value:4.6E-005, Ions matched by search engine: 15/164
Fragment match tolerance used for search: 20 mmu

Fragment Matches

Value Type: Theo. Mass [Da] | Ion Series | Neutral Losses | Precursor Ions

#1	a^+	a^{2+}	a^{3+}	b^+	b^{2+}	b^{3+}	Seq.	y^+	y^{2+}	y^{3+}	#2
1	44.04948	22.52838	15.35468	72.04440	36.52584	24.68632	A				15
2	158.09241	79.54984	53.36899	186.08733	93.54730	62.70063	N	1855.00328	928.00528	619.00594	14
3	286.15099	143.57913	96.05518	314.14591	157.57659	105.38682	Q	1740.96035	870.98381	580.99163	13
4	385.21941	193.11334	129.07799	413.21433	207.11080	138.40963	V	1612.90177	806.95452	538.30544	12
5	498.30348	249.65538	166.77268	526.29840	263.65284	176.10432	L	1513.83335	757.42031	505.28263	11
6	611.38755	306.19741	204.46737	639.38247	320.19487	213.79901	L	1400.74928	700.87828	467.58794	10
7	739.44613	370.22670	247.15356	767.44105	384.22416	256.48520	Q	1287.66521	644.33624	429.89325	9
8	853.48906	427.24817	285.16787	881.48398	441.24563	294.49951	N	1159.60663	580.30695	387.20706	8
9	1095.62696	548.31712	365.88050	1123.62187	562.31458	375.21214	K-GG	1045.56370	523.28549	349.19275	7
10	1208.71103	604.85915	403.57519	1236.70594	618.85661	412.90683	L	803.42580	402.21654	268.48012	6
11	1336.80600	668.90664	446.27352	1364.80091	682.90410	455.60516	K	690.34173	345.67450	230.78543	5
12	1465.84860	733.42794	489.28772	1493.84351	747.42540	498.61936	E	562.24676	281.62702	188.08710	4
13	1594.89120	797.94924	532.30192	1622.88611	811.94670	541.63356	E	433.20416	217.10572	145.07290	3
14	1723.93380	862.47054	575.31612	1751.92871	876.46800	584.64776	E	304.16156	152.58442	102.05870	2
15							R	175.11896	88.06312	59.04450	1

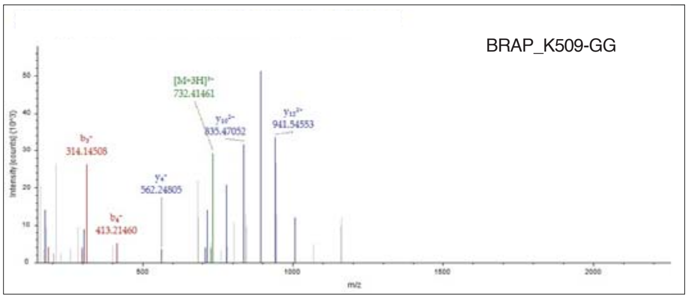

Peptide Summary

Sequence: ANQVLLQNKLKEEER, K11-LRGG (383.22810 Da)
Charge: +3, Monoisotopic m/z: 732.41327 Da (-0.11 mmu/-0.15 ppm), MH+: 2195.22525 Da, RT: 40.62 min,
Identified with: Mascot (v1.30); IonScore:26, Exp Value:4.3E-002, Ions matched by search engine: 9/164
Fragment match tolerance used for search: 20 mmu

Fragment Matches

Value Type: Theo. Mass [Da] | Ion Series | Neutral Losses | Precursor Ions

#1	a^+	a^{2+}	a^{3+}	b^+	b^{2+}	b^{3+}	Seq.	y^+	y^{2+}	y^{3+}	#2
1	44.04948	22.52838	15.35468	72.04440	36.52584	24.68632	A				15
2	158.09241	79.54984	53.36899	186.08733	93.54730	62.70063	N	2124.18845	1062.59787	708.73434	14
3	286.15099	143.57913	96.05518	314.14591	157.57659	105.38682	Q	2010.14552	1005.57640	670.72003	13
4	385.21941	193.11334	129.07799	413.21433	207.11080	138.40963	V	1882.08694	941.54711	628.03383	12
5	498.30348	249.65538	166.77268	526.29840	263.65284	176.10432	L	1783.01852	892.01290	595.01103	11
6	611.38755	306.19741	204.46737	639.38247	320.19487	213.79901	L	1669.93445	835.47087	557.31634	10
7	739.44613	370.22670	247.15356	767.44105	384.22416	256.48520	Q	1556.85038	778.93003	519.62165	9
8	853.48906	427.24817	285.16787	881.48398	441.24563	294.49951	N	1428.79180	714.89954	476.93545	8
9	981.58403	491.29565	327.86620	1009.57895	505.29311	337.19780	K	1314.74887	657.87808	438.92114	7
10	1094.66810	547.83769	365.56089	1122.66302	561.83515	374.89252	L	1186.65390	593.83059	396.22282	6
11	1605.99118	803.49923	536.00191	1633.98609	817.49668	545.33355	K-LRGG	1073.56983	537.28856	358.52811	5
12	1735.03378	868.02053	579.01611	1763.02869	882.01798	588.34775	E	562.24676	281.62702	188.08710	4
13	1864.07638	932.54183	622.03031	1892.07129	946.53928	631.36195	E	433.20416	217.10572	145.07290	3
14	1993.11898	997.06313	665.04451	2021.11389	1011.06058	674.37615	E	304.16156	152.58442	102.05870	2
15							R	175.11896	88.06312	59.04450	1

B

BRAP(124-569)-[M1-Ub3]n

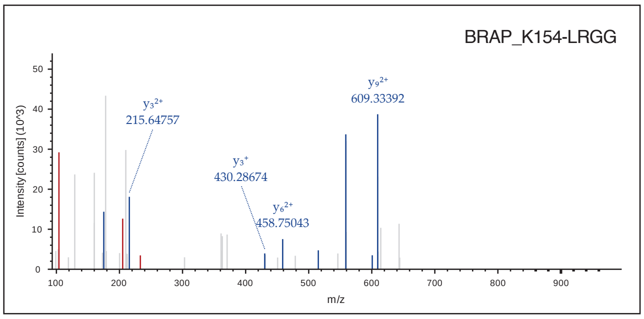

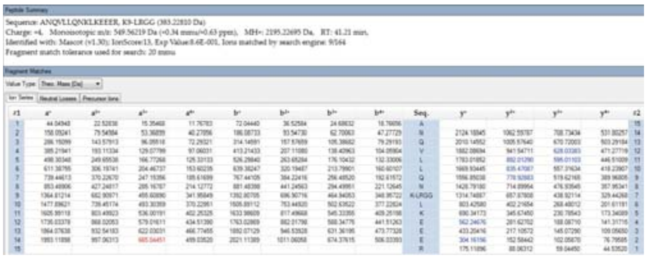

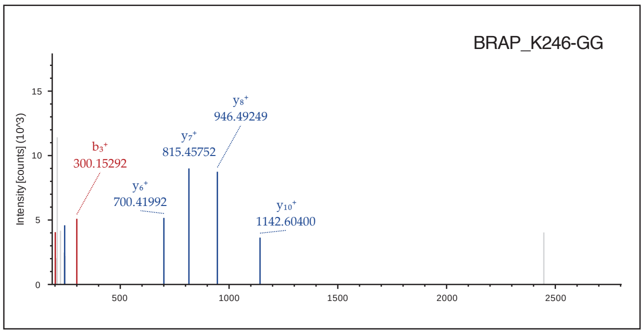

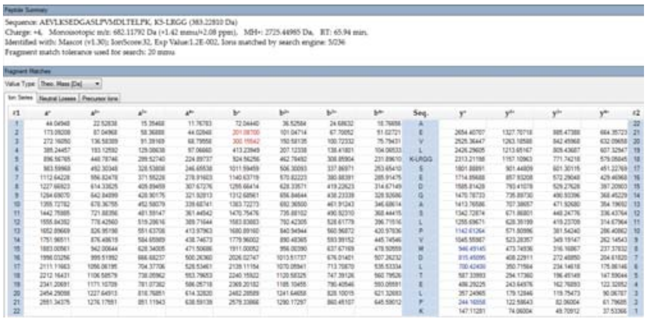

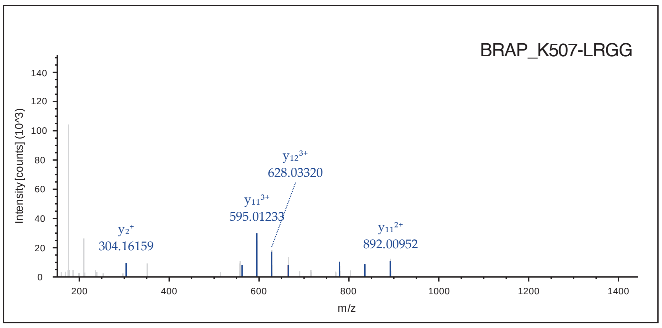

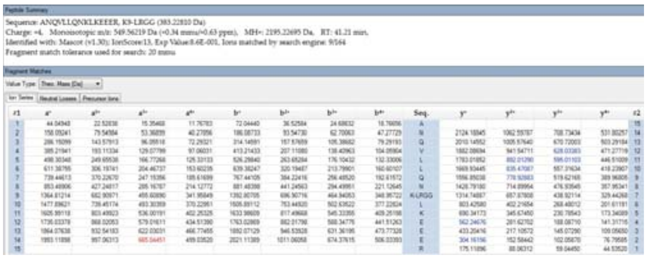

C

BRAP(124-569)-[M1-Ub4]n

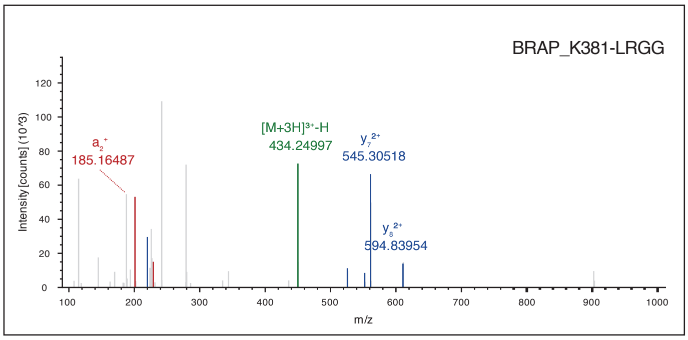

Peptide Summary

Sequence: LVASKTDGK, K5-LRGG (383.22810 Da)
Charge: +3, Monoisotopic m/z: 434.58963 Da (+0.24 mmu/+0.55 ppm), MH+: 1301.75434 Da, RT: 19.01 min,
Identified with: Mascot (v1.30); IonScore:11, Exp Value:1.9E+000, Ions matched by search engine: 6/80
Fragment match tolerance used for search: 20 mmu

Fragment Matches

Value Type: Theo. Mass [Da]

Ion Series | Neutral Losses | Precursor Ions

#1	a^+	a^{2+}	a^{3+}	b^+	b^{2+}	b^{3+}	Seq.	y^+	y^{2+}	y^{3+}	#2
1	86.09643	43.55185	29.37033	114.09135	57.54931	38.70197	L				9
2	185.16485	93.08606	62.39314	213.15977	107.08352	71.72477	V	1188.66955	594.83842	396.89470	8
3	256.20197	128.60462	86.07218	284.19689	142.60208	95.40381	A	1089.60113	545.30421	363.87190	7
4	343.23400	172.12064	115.08285	371.22892	186.11810	124.41449	S	1018.56401	509.78565	340.19286	6
5	854.55708	427.78218	285.52388	882.55199	441.77963	294.85551	K-LRGG	931.53198	466.26963	311.18218	5
6	955.60476	478.30602	319.20644	983.59967	492.30347	328.53807	T	420.20891	210.60809	140.74115	4
7	1070.63171	535.81949	357.54875	1098.62662	549.81695	366.88039	D	319.16123	160.08425	107.05859	3
8	1127.65318	564.33023	376.55591	1155.64809	578.32768	385.88755	G	204.13428	102.57078	68.71628	2
9							K	147.11281	74.06004	49.70912	1
